# MARK2 phosphorylates eIF2α in response to proteotoxic stress

**DOI:** 10.1371/journal.pbio.3001096

**Published:** 2021-03-11

**Authors:** Yu-Ning Lu, Sarah Kavianpour, Tao Zhang, Xumei Zhang, Dao Nguyen, Ravi Thombre, Lu He, Jiou Wang

**Affiliations:** 1 Department of Biochemistry and Molecular Biology, Bloomberg School of Public Health, Johns Hopkins University, Baltimore, Maryland, United States of America; 2 Department of Neuroscience, School of Medicine, Johns Hopkins University, Baltimore, Maryland, United States of America; Yale University, UNITED STATES

## Abstract

The regulation of protein synthesis is essential for maintaining cellular homeostasis, especially during stress responses, and its dysregulation could underlie the development of human diseases. The critical step during translation regulation is the phosphorylation of eukaryotic initiation factor 2 alpha (eIF2α). Here we report the identification of a direct kinase of eIF2α, microtubule affinity-regulating kinase 2 (MARK2), which phosphorylates eIF2α in response to proteotoxic stress. The activity of MARK2 was confirmed in the cells lacking the 4 previously known eIF2α kinases. MARK2 itself was found to be a substrate of protein kinase C delta (PKCδ), which serves as a sensor for protein misfolding stress through a dynamic interaction with heat shock protein 90 (HSP90). Both MARK2 and PKCδ are activated via phosphorylation in proteotoxicity-associated neurodegenerative mouse models and in human patients with amyotrophic lateral sclerosis (ALS). These results reveal a PKCδ-MARK2-eIF2α cascade that may play a critical role in cellular proteotoxic stress responses and human diseases.

## Abstract 2

The regulation of protein translation is vital for cellular stress responses and human diseases. This study identifies a new pathway that regulates the key step of translation initiation, with MARK2 directly phosphorylating eIF2α and acting downstream of PKCδ. This pathway is activated in conditions of cellular stress and in proteotoxicity-associated neurodegeneration.

## Introduction

To maintain a state of fitness during stress, cells have evolved exquisite stress response programs that sense potentially harmful situations and make the necessary adaptations at the molecular and cellular levels. Stress signaling pathways are frequently mediated by protein kinases and phosphorylation substrates, whose specificity is determined by their interactions with temporal and spatial regulations [1]. Proteins are responsible for most cellular functions, and the maintenance of protein homeostasis is required for the survival of cells, especially under stress conditions. A key regulation of protein homeostasis occurs at the level of protein synthesis or translation. The first step in translation requires eukaryotic initiation factor 2 (eIF2), which is regulated by phosphorylation of serine 51 (^51^S) of its alpha subunit (eIF2α), with increased phosphorylation resulting in global attenuation of the translation of most transcripts and enhanced translation of select transcripts encoding stress response-related proteins. The phosphorylation of eIF2α is the central step during the integrated stress response, which allows cells to react to various types of stimuli by regulating translation [2]. To date, 4 kinases have been found to phosphorylate eIF2α in response to various stressors: protein kinase R (PKR), activated by double-stranded RNA [3,4]; PKR-like ER-resident kinase (PERK), responding to endoplasmic reticulum (ER) stress [5]; heme-regulated eIF2α kinase (HRI), induced by low levels of heme [6,7]; and general control nonderepressible factor 2 kinase (GCN2), sensing amino acid deficiency [8]. Among them, PERK is capable of sensing protein misfolding as part of the unfolded protein response originating in the ER lumen [9,10]; HRI is expressed in an erythroid cell-specific manner and reported to be a cytosolic sensor of protein misfolding that controls innate immune signaling [11–13]. No other kinase has been identified that phosphorylates eIF2α and controls translation in response to protein unfolding stress.

Stresses associated with protein misfolding have formed a common theme in neurodegenerative diseases, including Alzheimer disease, Parkinson disease, Creutzfeldt–Jakob disease, Huntington disease, frontotemporal dementia (FTD), and amyotrophic lateral sclerosis (ALS) [14,15]. Among them, ALS is characterized by progressive motor neuron degeneration, with approximately 10% of cases inherited in families and both its familial and sporadic forms linked to diverse genetic mutations [16]. One of the central themes in ALS pathology is protein misfolding and aggregation. For example, proteinaceous inclusions that harbor misfolded proteins, including Cu/Zn superoxide dismutase (SOD1), have been found in both familial and sporadic ALS patients [17–20]. A large number of mutations in SOD1, responsible for 20% of all familial ALS, cause the protein to gain a heightened propensity to misfold and aggregate [21–25]. The contrast between wild-type (WT) and mutant SOD1 proteins, the former being highly stable and the latter prone to aggregation, makes SOD1 a sensitive molecular model for studying protein aggregation [26,27].

Here, we report the identification of microtubule affinity-regulating kinase 2 (MARK2), a serine/threonine kinase previously implicated in the regulation of microtubule stability [28,29], as a direct kinase of eIF2α under conditions of protein misfolding stress. MARK2 itself is a substrate of protein kinase C delta (PKCδ), a member of the PKC kinase family that has a conserved role in regulating cell polarity and signaling pathways [28–30]. Both MARK2 and PKCδ are phosphorylated under proteotoxic stress, and both kinases are required for the stress-induced phosphorylation of eIF2α. PKCδ serves as a sensor for protein misfolding stress through its dynamic interaction with the molecular chaperone HSP90. MARK2 and PKCδ are also activated in the nervous systems of mouse models of SOD1-linked ALS and in patients with ALS. These results reveal a cytosolic signaling pathway that regulates eIF2α phosphorylation and protein synthesis and may have important implications for our understanding of normal cellular stress responses and the pathogenic process in proteotoxicity-related neurodegenerative diseases.

## Results

### MARK2 is a direct kinase for eIF2α

**Fig 1:**
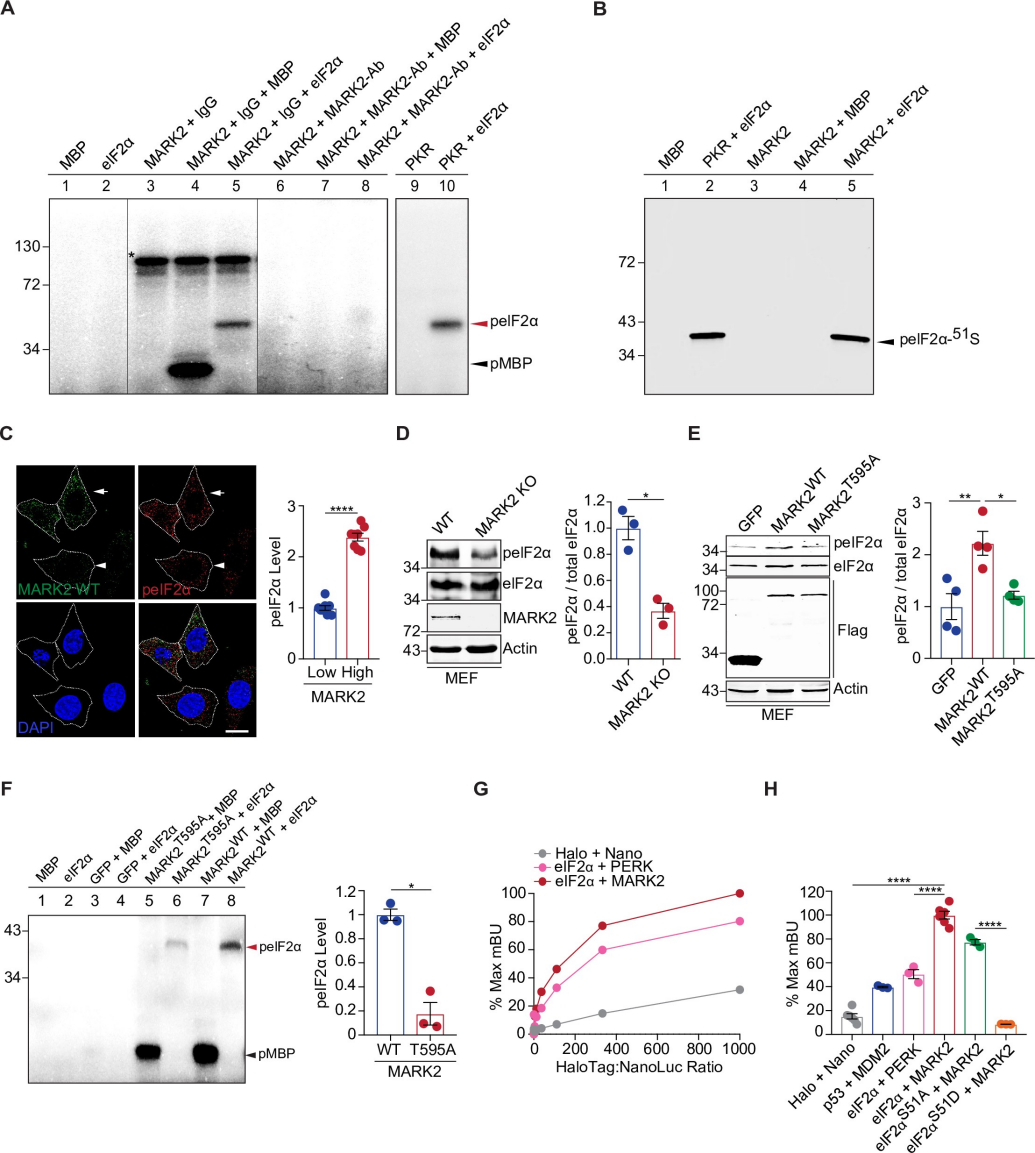
MARK2 is a direct kinase for eIF2α. (**A**) In vitro kinase assays using purified proteins and [γ-^32^P]-ATP show that MARK2 is a direct kinase for eIF2α. The MARK2-specific Ab, but not the IgG control, abolished the kinase activity. MBP was used as a positive control substrate for the kinase activity of MARK2. The asterisk indicates the autophosphorylated MARK2. (**B**) Immunoblot analyses of the reaction products from the in vitro kinase assay indicate that MARK 2 phosphorylates eIF2α at its serine 51 residue. PKR was used as a positive control kinase that phosphorylates eIF2α-^51^S. (**C**) Left: immunofluorescence of MARK2 (green) and endogenous phosphorylated eIF2α-^51^S (red) in MEFs. The arrow points to representative cells at the top with high MARK2 expression, and the arrowhead points to a representative cell at the bottom with low MARK2 expression. Right: quantification of the levels of phosphorylated eIF2α-^51^S in cells with high or low levels of MARK2 expression (*n* = 8). (**D**) The level of phosphorylated eIF2α-^51^S is significantly lower in MARK2 knockout MEFs than in WT control cells. Bar graph represents quantification of the immunoblot analysis (*n* = 3). (**E**) MEFs with elevated expression of MARK2^WT^ showed significantly increased levels of phosphorylated eIF2α-^51^S, as compared to MEFs expressing the mutant MARK2^T595A^. The bar graph represents quantification of the immunoblot analysis (*n* = 4). (**F**) In vitro kinase assays using [γ-^32^P]-ATP and MARK2 variants purified from HEK293 cells show that MARK2^WT^ is a direct kinase of eIF2α, but the T595A mutation significantly reduced its activity for phosphorylating eIF2α. The bar graph represents quantification of the radiograph analysis (*n* = 3). (**G**) The NanoBRET donor saturation assay indicates the specificity of the interaction between eIF2α and MARK2, as compared to the positive control PERK interaction with eIF2α and the nonspecific interaction between NanoLuc and HaloTag proteins. (**H**) The interaction between MARK2 and eIF2α variants, including its WT protein, phosphor-null mutant S51A, and phosphor-mimicking mutant S51D [*n* = 6 (Halo+Nano and eIF2α+MARK2), *n* = 3 (p53+MDM2, eIF2α+PERK, eIF2α^S51A^+MARK2, and eIF2α^S51D^+MARK2)]. Scale bar: 10 μm. Error bars represent ± SEM. *p ≤ 0.05; **p ≤ 0.01; ****p ≤ 0.0001. The data underlying the figure can be found in S1 Data. Ab, antibody; eIF2α, eukaryotic initiation factor 2 alpha; IgG, immunoglobulin G; KO, knockout; MARK2, microtubule affinity-regulating kinase 2; MBP, myelin basic protein; MEF, mouse embryonic fibroblast; PERK, PKR-like ER-resident kinase; PKR, protein kinase R; WT, wild-type.

Phosphorylation of eIF2α is a key step in the translational attenuation that occurs in response to a variety of stresses in mammalian cells [31]. To identify previously unrecognized eIF2α kinases, we searched a protein array dataset that suggested potential kinase and substrate relationships using microarrays composed of 4,191 unique human full-length proteins subjected to phosphorylation reactions with over 200 purified human kinases [32,33]. The protein array screen suggested at least 4 candidate kinases for eIF2α: protein tyrosine kinase 2 beta (PYK2), TTK protein kinase (TTK), bone morphogenetic protein receptor type 1A (BMPR1A), and MARK2. To determine which of these candidate kinases is capable of phosphorylating eIF2α, we performed in vitro kinase assays with radiolabeled ATP and proteins purified from Sf9 insect cells, including the eIF2α substrate and each of the 4 candidate kinases. Only MARK2 showed kinase activity, phosphorylating eIF2α in vitro (Fig 1A, lane 5 and S1A and S1B Fig). Myelin basic protein (MBP), a common substrate for diverse kinases, and PKR, a positive control kinase, were used to confirm that all the tested kinases were enzymatically active. Notably, the phosphorylation of eIF2α by MARK2 was completely blocked by a MARK2-specific antibody but not by an immunoglobulin G (IgG) control (Fig 1A, lane 8), confirming that the observed activity for the eIF2α kinase was specifically associated with the MARK2 protein.

To characterize the kinase activity of MARK2 toward eIF2α, we purified a series of WT and mutant MARK2 and eIF2α proteins using an *E*. *coli* expression system and performed in vitro enzyme kinetics analyses. The Kinase-Glo assay was used to measure kinase activities by quantifying ATP consumption via luminescent signals. First, by using MBP as a shared substrate, we observed that MARK2 and the positive control eIF2α kinase PKR showed similar reactivity as shown in Michaelis–Menten kinetics curves (S1C and S1D Fig). Additionally, we generated a kinase-dead mutant MARK2^KD^, which lacks the catalytic domain [34], as a negative control and confirmed the absence of kinase activity for this mutant (S1E Fig). Then, using the Km concentrations of MARK2 and PKR established above (S1C and S1D Fig), we studied the kinetics of their kinase activity toward eIF2α. We found that MARK2 exhibited a robust kinase activity for eIF2α comparable to that of PKR, as evidenced by the similarity of Km and Vmax values in Michaelis–Menten kinetics curves between the 2 sets of reactions (S1F and S1G Fig). As expected, the kinase-dead MARK2^KD^ mutant did not show activity toward eIF2α (S1H Fig).

Translational control via the phosphorylation of eIF2α at serine 51 is a point of convergence for integrated stress response pathways [31]. Using a phosphorylation-dependent antibody against phospho-eIF2α-^51^S, we showed that the radiolabeled phospho-eIF2α signal seen in the kinase assay with MARK2 was positively recognized by the antibody against the phosphorylated eIF2α-^51^S (Fig 1B). To further validate the phosphorylation of eIF2α by MARK2 at serine 51, we purified the phosphor-null mutant eIF2α^S51A^. Using the in vitro kinase assay based on ATP radiolabeling and gel electrophoresis, we confirmed that eIF2α^S51A^ was not phosphorylated, when compared to the WT form of the substrate, by either PKR or MARK2 (S1I Fig). The kinase-dead MARK2^KD^ mutant did not show activity toward eIF2α^WT^ or eIF2α^S51A^ (S1I Fig). Consistently, in the kinetics analysis, the eIF2α^S51A^ mutant protein showed substantially lower reactivity for PKR or MARK2 than WT eIF2α, as evidenced by the curve slopes and Km values (S1F and S1G Fig). Together, these results demonstrate that MARK2 directly phosphorylates eIF2α at serine 51.

### MARK2 is a kinase for eIF2α in mammalian cells

To study the physiologically relevant kinase activity of MARK2 in vivo, we analyzed the kinase activity of MARK2 on eIF2α in mammalian cells. Using mouse embryonic fibroblasts (MEFs), we compared cells expressing different levels of MARK2 for their correlation with the levels of phosphorylated eIF2α-^51^S: The cells with relatively higher levels of cytoplasmic MARK2 showed higher levels of phosphorylated eIF2α-^51^S, whereas the neighboring cells with less MARK2 showed lower levels of phosphorylated eIF2α-^51^S, as demonstrated by immunofluorescent staining for both MARK2 and eIF2α (Fig 1C), suggesting that MARK2 positively regulates eIF2α phosphorylation in the cells. Next, we examined MARK2-mediated regulation of eIF2α phosphorylation by immunoblot analysis. Using MEFs in which the MARK2 locus was disrupted by the deletion of exons 2 to 4 [35], we found that the absence of MARK2 resulted in a significant reduction in the level of phosphorylated eIF2α-^51^S, without any detectable change in the level of total eIF2α protein (Fig 1D). This finding was validated in human HAP1 cells in which the MARK2 gene was disrupted with a CRISPR-engineered premature stop codon in exon 2 (S2A Fig). Consistent with the result in MEFs, the human MARK2 knockout cells showed substantially lower levels of phosphorylated eIF2α-^51^S than did the WT control cells (S3A Fig). Conversely, in MEFs that stably expressed elevated levels of MARK2, the phosphorylation of eIF2α-^51^S was significantly increased, without changing the level of total eIF2α protein (Fig 1E). The specificity of the antibody against phosphorylated eIF2α-^51^S was verified in an eIF2α^S51A^ knockin mutant MEF line, in which the S51A mutation abolished the immunoblot signal of phosphorylated eIF2α-^51^S (S4A Fig). Consistent with the regulation of eIF2α phosphorylation by MARK2, we observed that elevated expression of MARK2 led to attenuation of global translation, as shown by the reduction of the protein synthesis rate measured by either puromycin incorporation or ^35^S-Methionine/Cysteine labeling (S4B and S4C Fig). Together, these results demonstrate that MARK2 promotes the phosphorylation of eIF2α-^51^S and suppresses translation in the cell.

MARK2 itself is phosphorylated, and the most studied phosphorylation site is at its threonine 595 (^595^T) residue [28,29]. To test whether the kinase activity of MARK2 on eIF2α was dependent on its phosphorylation at threonine 595, the threonine 595 residue was mutated to alanine (A), and stable MEF lines were generated expressing the MARK2^T595A^ mutant. In the MEF cells, the MARK2^T595A^ mutant did not exhibit the ability to promote the phosphorylation of eIF2α-^51^S when compared to the MARK2^WT^ (Fig 1E). Unlike its WT counterpart, the MARK2_T595A_ mutant was much less effective in inducing a reduction in the global protein synthesis rate (S4B and S4C Fig). To confirm this result, we performed in vitro kinase activity assays using WT and mutant MARK2 proteins purified from HEK293 cells. MARK2^T595A^ showed a much lower level of eIF2α kinase activity than did MARK2^WT^ in the radiolabeled eIF2α phosphorylation assay (Fig 1F). These results indicate that the MARK2 kinase activity for eIF2α requires the phosphorylation of MARK2 at threonine 595.

To confirm the direct interaction between MARK2 and eIF2α in live cells, we employed a proximity-based protein–protein interaction assay, NanoBRET, based on bioluminescence resonance energy transfer (BRET), to study the dynamic interaction between MARK2 and eIF2α in natural cellular environment. MARK2 was fused with the energy donor NanoLuc, and eIF2α was fused with the energy acceptor HaloTag; an interaction between MARK2 and eIF2α would bring the energy donor and acceptor into proximity and give rise to detectable BRET signals (S2G Fig). When the MARK2 and eIF2α fusion proteins were coexpressed in HEK293 cells, a strong BRET signal was detected, while the unfused NanoLuc and HaloTag proteins served as the negative control and showed much lower background BRET signals. Donor saturation assays were performed to monitor the kinetics of the direct interaction between MARK2 and eIF2α in the live cells. The MARK2 and eIF2α pair showed a hyperbolic curve, indicating that the energy transfer value reached a maximum when all the donors were saturated with the acceptors (Fig 1G). Interestingly, the interaction between MARK2 and eIF2α indicated by the BRET signal of the pair was stronger than that between the known kinase PERK and eIF2α, which itself was stronger than the nonspecific interaction between unfused NanoLuc and HaloTag, in the donor saturation assay (Fig 1G). Moreover, the interaction between MARK2 and eIF2α was stronger than that of another positive control interacting pair, p53 and MDM2, in the quantitative NanoBRET assay (Fig 1H). To determine how phosphorylation of eIF2α at the serine 51 site affects its interaction with MARK2, we generated the phosphor-null and phosphor-mimicking mutants S51A and S51D, respectively, for eIF2α and subjected them to the NanoBRET assay. Whereas the eIF2α^S51A^ mutant retained much of the interaction with MARK2, the eIF2α^S51D^ mutant showed no interaction with MARK2 (Fig 1H), suggesting that the phosphorylation event diminishes the interaction between MARK2 and eIF2α. Collectively, these results support the notion that MARK2 is a direct and specific kinase of eIF2α at its serine 51 site both in vitro and in vivo.

### MARK2 mediates eIF2α phosphorylation independently of previously known kinases

**Fig 2:**
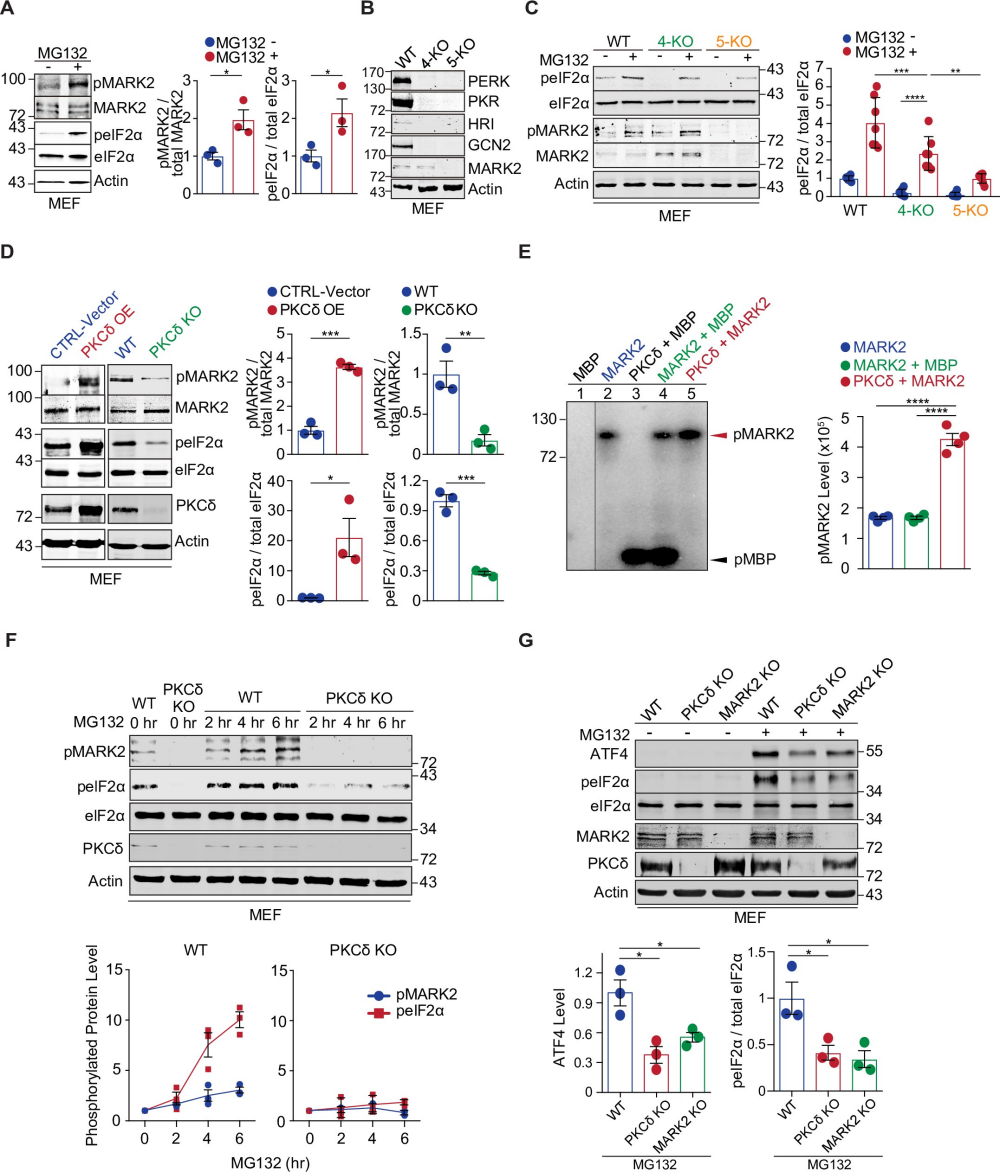
A PKCδ-MARK2-eIF2α signaling cascade in response to protein misfolding stress. (**A**) Immunoblot analyses of MEFs treated with MG132 indicate that the stress increases the level of phosphorylated MARK2-^595^T and eIF2α-^51^S. Bar graphs represent the quantification of the immunoblots (*n* = 3). (**B**) PERK, PKR, HRI, GCN2, and MARK2 proteins were analyzed by immunoblotting in the 4-KO and 5-KO MEFs as compared to the WT MEFs. (**C**) Immunoblot analyses of WT, 4-KO (PERK, GCN2, HRI, and PKR), and 5-KO (PERK, PKR, HRI, GCN2, and MARK2) MEFs treated with MG132 indicate that eIF2α-^51^S is still phosphorylated in response to the stress in the 4-KO MEFs. The levels of phosphorylated eIF2α-^51^S in the 5-KO cells are significantly lower than those in the 4-KO MEFs. Bar graphs represent the quantification of the immunoblots (*n* = 7; 5-KO without MG132 vs. 5-KO with MG132 *p* = 0.1893). (**D**) PKCδ controls the phosphorylation levels of MARK2-^595^T and eIF2α-^51^S, as indicated by immunoblot analyses of PKCδ KO MEFs and the opposite MEFs that stably express elevated levels of PKCδ (OE). Bar graphs represent the quantification of the immunoblots (*n* = 3). (**E**) In vitro kinase assays demonstrate that PKCδ phosphorylates MARK2. MARK2 exhibits autophosphorylation (lane 2). MBP was used as a positive control substrate. The presence of PKCδ significantly increases the phosphorylation of MARK2 (lane 5). As a candidate substrate, the MARK2 protein level in lane 5 was only half of the MARK2 levels in lanes 2 and 4. The bar graph shows the quantification of the in vitro kinase assays (*n* = 4). (**F**) Immunoblot analyses of PKCδ KO and control MEFs treated with the proteasome inhibitor, MG132 (500 nM), for 2, 4, or 6 h indicate that the absence of PKCδ abolishes stress-induced phosphorylation of MARK2. Line graphs show the quantification of the immunoblots (*n* = 3). (**G**) Correlative changes in the protein levels of ATF4 and phosphorylated eIF2α in PKCδ or MARK2 KO and control MEFs, under proteotoxic stress induced by MG132. Bar graph represents quantification of the immunoblots (*n* = 3). Error bars represent ± SEM. *p ≤ 0.05; **p ≤ 0.01; ***p ≤ 0.001; ****p ≤ 0.0001. The data underlying the figure can be found in S1 Data. eIF2α, eukaryotic initiation factor 2 alpha; GCN2, general control nonderepressible factor 2 kinase; HRI, heme-regulated eIF2α kinase; KO, knockout; MARK2, microtubule affinity-regulating kinase 2; MBP, myelin basic protein; MEF, mouse embryonic fibroblast; PERK, PKR-like ER-resident kinase; PKCδ, protein kinase C delta; PKR, protein kinase R; WT, wild-type.

Since the phosphorylation of MARK2 at threonine 595 is required for its positive regulation of eIF2α phosphorylation (Fig 1E), we asked whether this form of phosphorylated MARK2 is regulated upon cytosolic protein misfolding stress. To induce the protein misfolding stress, we treated MEF cells with the proteasome inhibitor MG132, and it elicited a substantial increase in the levels of phosphorylated MARK2-^595^T as well as a corresponding increase in the levels of phosphorylated eIF2α-^51^S (Fig 2A). In a time course study using the treatment with 500 nM MG132 for 24 h, the proteotoxic stressor led to a time-dependent increase in the levels of phosphorylated MARK2-^595^T, together with concomitantly increased levels of phosphorylated eIF2α-^51^S (S3B Fig). These data suggest that the MARK2-eIF2α signaling pathway is activated in response to the proteotoxic stress.

Next we tested the activation of MARK2 by proteotoxic stress in the absence of PERK, HRI, PKR, or GCN2. In all 4 types of knockout MEFs lacking each of the 4 kinases, MARK2 was activated under the MG132-induced stress, as indicated by the increased phosphorylation at its threonine 595 site (S3C–S3F Fig). Accordingly, the phosphorylation of eIF2α-^51^S was also significantly increased (S3C–S3F Fig). These data indicate that the activation of the MARK2-eIF2α pathway by the protein misfolding stress does not require any of the 4 previously known eIF2α kinases.

To further demonstrate that MARK2 alone is sufficient to promote the phosphorylation of eIF2α in the absence of all 4 previously known eIF2α kinases, we used multiplex CRISPR-Cas9 gene editing to knock out PERK, GCN2, and HRI in an existing PKR-knockout MEF line [36], creating 4-KO MEF lines (Fig 2B and S2C–S2F Fig). Next, we applied the proteasome inhibitor MG132 to the 4-KO MEF cells and found that the stress response, as indicated by eIF2α phosphorylation, was still intact. The phosphorylation of eIF2α-^51^S induced by the proteotoxic stress was lower in the 4-KO MEFs than in the WT MEFs (Fig 2C); however, when compared to the unstressed cells, the phosphorylation of eIF2α in the absence of all 4 previously established kinases remained clearly detectable (Fig 2C). In the 4-KO MEFs, the stress-induced change in the levels of phosphorylated eIF2α-^51^S was correlated with the increase in the level of MARK2-^595^T (Fig 2C). To test whether MARK2 mediates the phosphorylation of eIF2α, we created independent 5-KO MEF lines by knocking out MARK2 in the 4-KO MEFs (Fig 2C). When the 5-KO MEFs were treated with MG132, the phosphorylation of eIF2α was still enhanced, but the increase was significantly less than that in the 4-KO MEFs (Fig 2C), indicating that MARK2 is capable of promoting eIF2α phosphorylation independently of the 4 previously known kinases. The condition of MG132 treatment at 20 μM for 4 h was chosen for optimal induction of eIF2α phosphorylation in the analysis of these MEFs (S4D Fig). To test whether activation of MARK2 can also be induced by other types of stress, we subjected the cells to oxidative stress such as sodium arsenite treatment or ER stress such as tunicamycin treatment. The sodium arsenite treatment, known to cause protein damages throughout the cell, was able to induce the phosphorylation of MARK2-^595^T and eIF2α-^51^S in both WT MEFs and those lacking the 4 previously known kinases (S4E Fig), consistent with the activation of MARK2 by proteotoxic stress. In comparison, the tunicamycin treatment, known to activate PERK, has no effect on the phosphorylation of MARK2-^595^T, induced the phosphorylation of eIF2α-^51^S in WT MEFs but not in the cells lacking the 4 previously known kinases including PERK (S4F Fig), consistent with the notion that MARK2 and ER stress act via independent pathways to regulate eIF2α phosphorylation.

It was reported that the PKR KO MEF line that we used to generate the 4-KO and 5-KO cells expresses a remnant C-terminal fragment of the PKR protein [36,37]. We used CRISPR editing to create additional deletion mutations in the PKR gene in the exiting 4-KO and 5-KO cells (S2F Fig) and confirmed the disruption of the remnant C-terminal fragment, as validated by the absence of its expression under interferon-α stimulation, in the newly generated 4-KO′ and 5-KO′ MEF lines (S4G Fig). Examination of the phosphorylation of eIF2α-^51^S induced by MG132 in the 4-KO′ and 5-KO′ MEFs showed similar results as those in the 4-KO and 5-KO cells (S4H Fig), confirming the MARK2-dependent phosphorylation of eIF2α in the absence of the 4 previously known kinases.

Furthermore, we examined the activity of protein phosphatase 1 (PP1), which catalyzes the dephosphorylation of eIF2α [38]. PP1α is phosphorylated at threonine 320 (^320^T), which inhibits its phosphatase activity [39]. We observed a decrease in the phosphorylation of PP1α-^320^T in 4-KO′ and 5-KO′ MEFs compared to WT MEFs, suggesting that the activation of PP1 contributes to the decrease in eIF2α phosphorylation in the 4-KO′ and 5-KO′ MEFs (S4H Fig). Additionally, there was a trend for an increase in the phosphorylation of PP1α-^320^T in the 5-KO′ MEFs compared to the 4-KO′ MEFs (S4H Fig). Since the reduced activity of PP1 in the 5-KO′ MEFs as compared to that in the 4-KO′ MEFs would enhance eIF2α phosphorylation in the 5-KO′ MEFs, the significant decrease in the phosphorylation of eIF2α-^51^S observed in the 5-KO′ MEFs as compared to the 4-KO′ MEFs, as a result of the loss of MARK2, supports the notion that MARK2 plays a major role in directly promoting eIF2α phosphorylation.

### A PKCδ-MARK2-eIF2α signaling pathway in response to protein misfolding stress

To understand the regulation of MARK2 activation under conditions of protein misfolding, we sought to identify the upstream kinase that is responsible for activating MARK2 in response to the stress conditions. In the course of our studies, we tested PKCδ, a member of the PKC family, as a potential kinase of MARK2, because PKCδ was shown to be activated during stress responses, and another member of the PKC family was previously reported to phosphorylate MARK2 in the regulation of cell polarity [28,29]. First, we generated MEF cell lines that stably expressed PKCδ and observed a substantial increase in the level of phosphorylated MARK2-^595^T, suggestive of the activation of MARK2 when the PKCδ level was elevated (Fig 2D, left). The phosphorylation of eIF2α-^51^S was increased in correlation with that of MARK2-^595^T. Conversely, in PKCδ-knockout MEFs, the signals for phosphorylated MARK2, and accordingly that of eIF2α, were abolished (Fig 2D, right). As controls, the total levels of MARK2 and eIF2α protein were not altered. Next, we asked whether PKCδ would exhibit any direct kinase activity toward MARK2. Using in vitro kinase assays with purified proteins, we found that PKCδ significantly increased the level of phosphorylated MARK2, despite a background of autophosphorylation of MARK2, indicating that PKCδ has intrinsic kinase activity for MARK2 (Fig 2E). In comparison, PKCδ did not show any kinase activity toward eIF2α in the in vitro kinase assay, indicating that PKCδ is not a direct kinase of eIF2α (S1B Fig, lane 8). These results therefore point to a PKCδ-MARK2-eIF2α signaling pathway that operates in the cell.

To determine whether PKCδ is required for the activation of MARK2-eIF2α signaling downstream, we applied MG132-induced proteotoxic stress to MEF cells with or without PKCδ. In the WT MEFs, MG132 elicited a robust up-regulation of phosphorylated MARK2-^595^T, reflective of increased MARK2 kinase activity; concomitantly, we observed a parallel increase in the phosphorylation of eIF2α-^51^S (Fig 2F). In contrast, in the PKCδ-knockout MEFs, we saw a substantially diminished increase in the levels of phosphorylated MARK2-^595^T or eIF2α-^51^S in response to MG132, indicating inhibition of MARK2 kinase activity toward eIF2α (Fig 2F). Furthermore, consistent with the activation of the MARK2-eIF2α pathway by MG132 treatment in the knockout MEFs lacking each of the 4 known kinases of eIF2α (PERK, HRI, PKR, and GCN2), PKCδ was also activated, as demonstrated by the phosphorylation of its threonine 505 (^505^T) site [40] (S3C–S3F Fig). This result indicated that the PKCδ-MARK2-eIF2α signaling is independent of the previously known eIF2α kinases.

Although the phosphorylation of eIF2α leads to attenuation of the translation of most transcripts, it also increases the translation of specific mRNAs such as activating transcription factor 4 (ATF4) [41] as part of the stress response. Indeed, treatment of MEF cells with MG132, which induces proteotoxicity, increased the level of the ATF4 protein (Fig 2G). However, the increase in the ATF4 level was significantly reduced in knockout MEFs lacking PKCδ or MARK2 (Fig 2G). Accordingly, the levels of eIF2α phosphorylation were significantly reduced in the absence of PKCδ or MARK2 (Fig 2G). These observations demonstrate that PKCδ and MARK2 are major positive regulators of eIF2α phosphorylation during MG132-induced proteotoxic stress, providing evidence for a PKCδ-MARK2-eIF2α signaling cascade.

### HSP90 interacts with PKCδ and mediates stress-dependent activation of PKCδ

**Fig 3:**
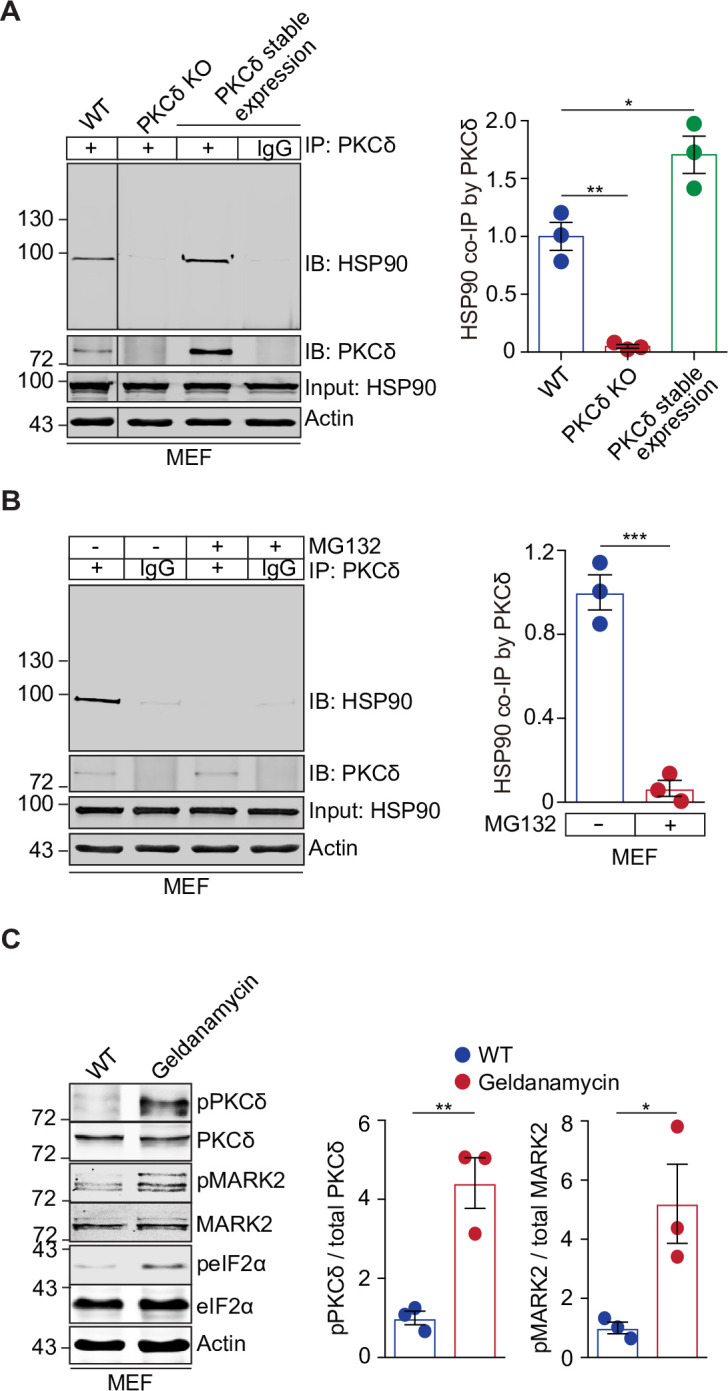
HSP90 interacts with PKCδ and mediates proteotoxicity-induced activation of the PKCδ-MARK2-eIF2α signaling pathway. (**A**) Coimmunoprecipitation analyses of HSP90 as pulled down by the anti-PKCδ antibody in control MEF cells, as compared to those stably expressing PKCδ or completely lacking PKCδ. IgG was used as a control for the anti-PKCδ antibody. The results indicate that PKCδ specifically interacts with HSP90 in a manner dependent on the levels of PKCδ (*n* = 3). (**B**) Coimmunoprecipitation analyses of HSP90 as pulled down by the anti-PKCδ antibody in MEFs cells with or without treatment with MG132 indicate that the proteotoxic stress abolished the interaction between PKCδ and HSP90 (*n* = 3). (**C**) Immunoblot analyses of MEFs treated with geldanamycin (2 μM, 1 h) versus the DMSO control indicate that inhibition of HSP90 substantially increased the phosphorylation of PKCδ-^505^T, MARK2-^595^T, and eIF2α-^51^S. Bar graphs represent the quantification of the immunoblots (*n* = 3). Error bars represent ± SEM. *p ≤ 0.05; **p ≤ 0.01; ***p ≤ 0.001. The data underlying the figure can be found in S1 Data. eIF2α, eukaryotic initiation factor 2 alpha; HSP90, heat shock protein 90; IgG, immunoglobulin G; KO, knockout; MARK2, microtubule affinity-regulating kinase 2; MEF, mouse embryonic fibroblast; PKCδ, protein kinase C delta; WT, wild-type.

We next investigated how the PKCδ-MARK2-eIF2α signaling pathway senses protein misfolding stress. Since misfolded proteins, including SOD1, are prone to interact with molecular chaperone proteins [26,42], we reasoned that the upstream regulator PKCδ could potentially interact with a molecular chaperone and that disruption of this interaction by misfolded proteins during proteotoxic stress might be a mechanism to account for the activation of the PKCδ kinase. We searched a protein–protein interaction database [43] and identified several candidate interactors of PKCδ, including HSP90, HSP90α, and HSP70. To evaluate potential interactions between PKCδ and these molecular chaperones, we performed coimmunoprecipitation experiments with endogenous proteins in MEF cells. After immunoprecipitation of PKCδ, only HSP90 was pulled down by PKCδ (Fig 3A). When the level of PKCδ protein was elevated via stable expression in MEF cells, more HSP90 protein could be coimmunoprecipitated by PKCδ. Conversely, depletion of PKCδ from MEFs or use of an IgG control instead of the anti-PKCδ antibody abolished the coimmunoprecipitation of HSP90, confirming the specific interaction between PKCδ and HSP90 (Fig 3A). In comparison, we did not detect an interaction between PKCδ and HSP70 in the same immunoprecipitation assays using either WT MEFs or cells with stable expression or depletion of PKCδ (S5A Fig). To test whether increased levels of misfolded proteins could disrupt the interaction between PKCδ and HSP90, we performed coimmunoprecipitation assays in MEF cells treated with MG132. We found that the MG132-induced proteotoxic stress completely abolished the interaction between PKCδ and HSP90, suggesting that the accumulated misfolded proteins compete for the binding of HSP90 and release PKCδ from the complex (Fig 3B).

To determine whether HSP90 regulates the activation of PKCδ, we tested the effect in MEF cells of a specific inhibitor of HSP90, geldanamycin [44], on the phosphorylation of PKCδ at residue threonine 505, which is required for the kinase activity of PKCδ [40]. We found that this HSP90 inhibitor substantially enhanced the phosphorylation of PKCδ-^505^T (Fig 3C). Concomitant with the activation of PKCδ, there was an increase in the phosphorylation of MARK2-^595^T and eIF2α-^51^S in the cells treated with the HSP90 inhibitor (Fig 3C). Similar results were observed for the increase in the phosphorylation of PKCδ-^505^T, MARK2-^595^T, and eIF2α-^51^S, when HSP90 was genetically knocked down in MEFs (S5B Fig). These data demonstrate that inhibition of HSP90 results in the activation of the PKCδ-MARK2-eIF2α signaling pathway. Collectively, these results suggest that PKCδ and HSP90 form a complex that can sense protein misfolding stress through the intrinsic affinity of HSP90 for misfolded proteins and thereby regulate the activation of PKCδ-MARK2-eIF2α signaling.

### Misfolded SOD1 induces PKCδ-MARK2-eIF2α signaling

**Fig 4:**
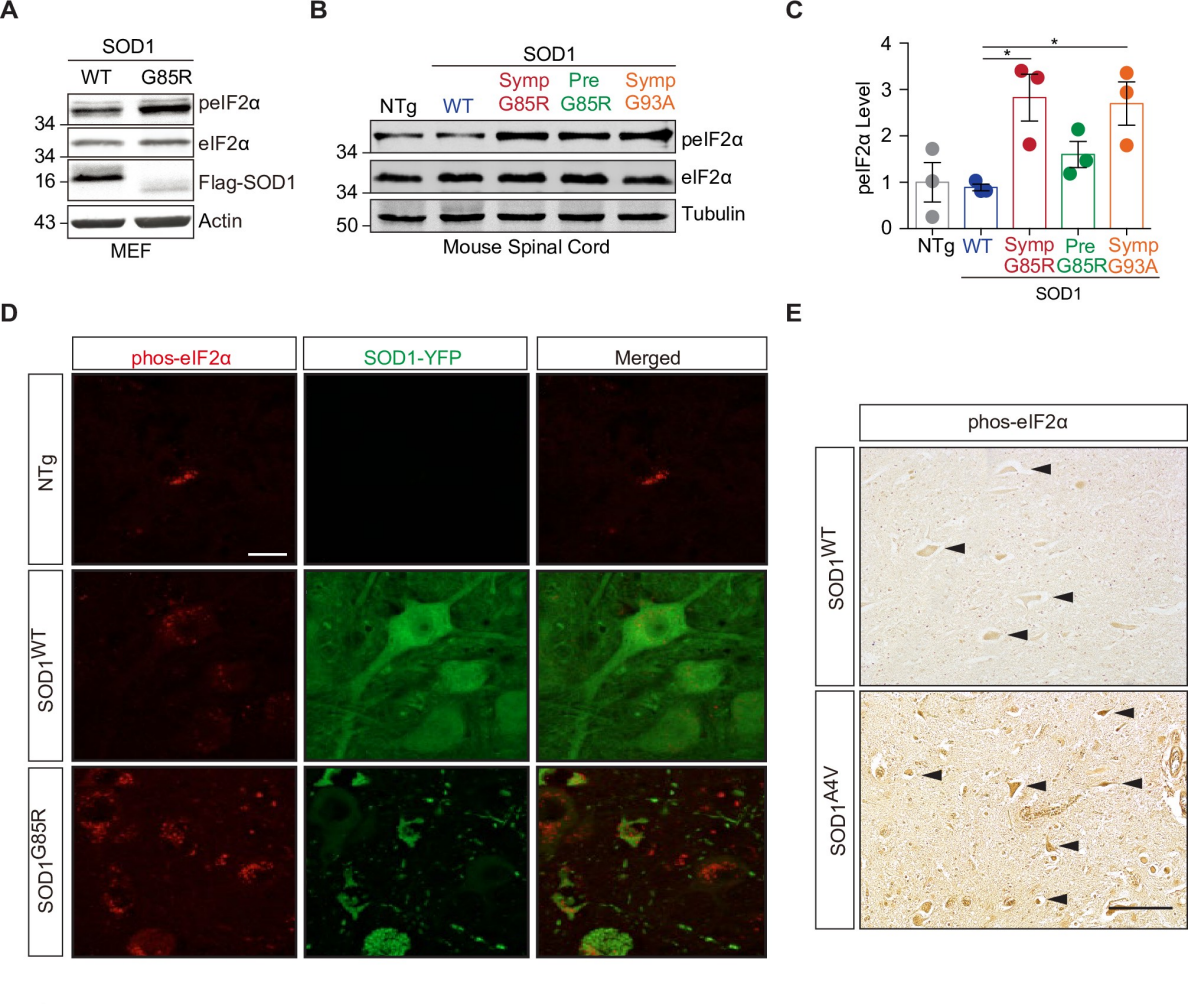
The expression of misfolded mutant SOD1 leads to phosphorylation of eIF2α in mammals. (**A**) The phosphorylation of eIF2α was increased upon expression of SOD1^G85R^ in MEFs as compared to the SOD1^WT^ control. The SOD1 proteins are Flag-tagged, and immunoblot analyses are shown. (**B**) Immunoblot analyses of spinal cord lysates from NTg (>10 months), SOD1^WT-YFP^ (nonsymptomatic, >10 months), SOD1^G85R-YFP^ (presymptomatic at 7 months and symptomatic at 8 months), and SOD1^G93A^ (symptomatic at 6 months) transgenic mice show an increase in phosphorylation of eIF2α at its serine 51 residue, occurring in a mutant SOD1- and symptom-dependent manner. (**C**) Quantification of immunoblots in (B) (*n* = 3 independent sets of mice). (**D**) Immunostaining in the spinal cords from NTg, SOD1^WT-YFP^ (>10 months), and SOD1^G85R-YFP^ (symptomatic at 8 months) mice demonstrates increased phosphorylation of eIF2α-^51^S in the symptomatic mutant mice. Scale bar: 25 μm. (**E**) Immunostaining in the spinal cord from an SOD1^A4V^-ALS patient and an age-matched human control indicates increased phosphorylation of eIF2α-^51^S in the patient’s tissue. Scale bar: 100 μm. Error bars represent ± SEM. *p ≤ 0.05. The data underlying the figure can be found in S1 Data. eIF2α, eukaryotic initiation factor 2 alpha; MEF, mouse embryonic fibroblast; NTg, nontransgenic control; Pre, presymptomatic; SOD1, Cu/Zn superoxide dismutase; Symp, symptomatic; WT, wild-type.

ALS-linked mutant SOD1 proteins, including the G85R variant, are prone to misfolding and aggregation, providing a sensitive molecular model for studying proteotoxicity [26,45]. Next, we asked whether the misfolded mutant SOD1^G85R^ affects the phosphorylation of eIF2α. We generated stable MEF lines that express SOD1^WT^ or SOD1^G85R^ in an inducible manner. Upon induction, we found that SOD1^G85R^ caused a marked increase in the phosphorylation of eIF2α-^51^S when compared to the SOD1^WT^ control (Fig 4A). We then used a set of transgenic mouse models expressing WT or disease-associated SOD1 variants in order to investigate the regulation of eIF2α phosphorylation. We have previously shown that SOD1^G85R-YFP^ causes age-dependent motor neuron degeneration in mice, with a marked accumulation of protein aggregates upon disease onset; in contrast, control SOD1^WT-YFP^ mice are free of disease symptoms and protein aggregation pathology [26]. When compared to nontransgenic or SOD1^WT-YFP^ controls, SOD1^G85R-YFP^ mice at the presymptomatic stage showed a moderate increase in eIF2α-^51^S phosphorylation in the spinal cords, as measured by immunoblotting; however, in the symptomatic SOD1^G85R-YFP^ mice, the phosphorylation of eIF2α-^51^S was remarkably increased (Fig 4B and 4C). The increase in eIF2α-^51^S phosphorylation in SOD1^G85R-YFP^ motor neurons, which harbor pronounced cytoplasmic aggregates in affected mice, was confirmed by immunofluorescent staining of spinal cord sections (Fig 4D). A similar increase in eIF2α-^51^S phosphorylation was observed and quantified by immunoblotting in transgenic mice expressing another disease-associated mutant, SOD1^G93A^, at the symptomatic stage (Fig 4B and 4C). Furthermore, in a human ALS patient carrying the SOD1^A4V^ mutation, we found by immunohistochemical analysis that the eIF2α phosphorylation was also markedly increased in spinal cord motor neurons (Fig 4E). These results suggest that eIF2α phosphorylation and subsequent translational regulation are a pathological consequence of mutant SOD1 in mammalian systems.

### Increased phosphorylation of PKCδ-^505^T and MARK2-^595^T in ALS mice and patients

**Fig 5:**
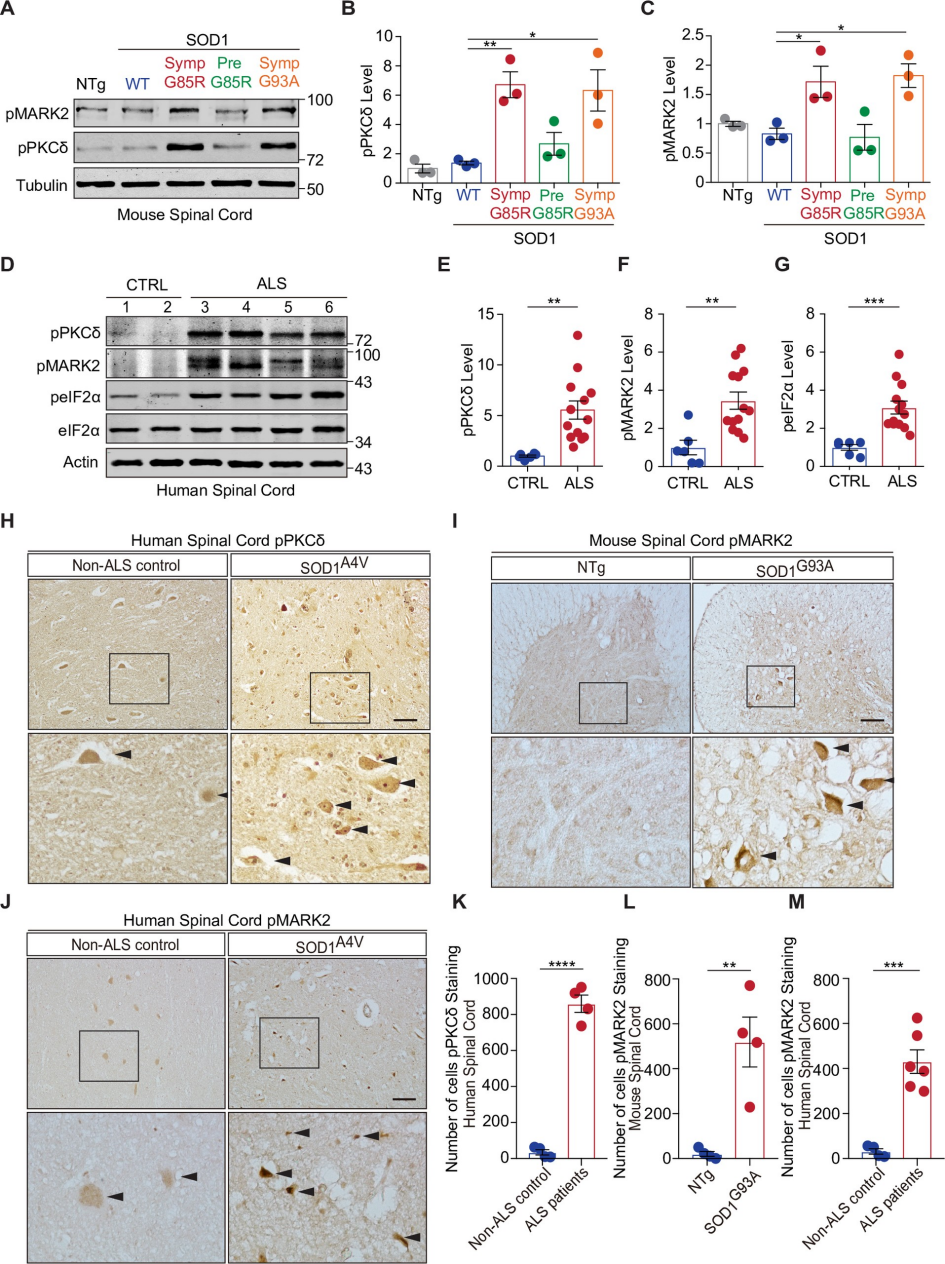
The PKCδ-MARK2-eIF2α signaling pathway is altered in ALS mouse models and patients. (**A**) Immunoblot analyses of spinal cord lysates from NTg, SOD1^WT-YFP^, presymptomatic and symptomatic SOD1^G85R-YFP^, and SOD1^G93A^ transgenic mice show an increase in phosphorylation of PKCδ at its threonine 505 residue, occurring in a mutant SOD1- and symptom-dependent manner. (**B**, **C**) Bar graph represents the quantification of the immunoblots in (A) (*n* = 3 sets of mice). (**D**) Representative immunoblot analyses of PKCδ, MARK2-^595^T, and eIF2α in the spinal cord tissues from ALS patients and non-ALS controls, indicating that increased phosphorylation of PKCδ-^505^T, MARK2-^595^T, and eIF2α-^51^S is a general phenotype in patient tissues. (**E**–**G**) Bar graph represents the quantification of the immunoblots in (D) (ALS: *n* = 13; CTRL: *n* = 6). (**H**) Representative immunostaining in the spinal cords from a symptomatic SOD1^G93A^ mouse and an age-matched control shows increased phosphorylation of MARK2-^595^T in the mutant animal. (**I**) Immunostaining in the spinal cord from an SOD1^A4V^-ALS patient and an age-matched control indicates increased phosphorylation of PKCδ-^505^T in the patient’s tissue. (**J**) Representative immunostaining in the spinal cord from an SOD1^A4V^-ALS patient and an age-matched human control indicates increased phosphorylation of MARK2-^595^T in the patient’s tissue. (**K**) Quantification of the number of cells with elevated levels of phosphorylated PKCδ in (H) (*n* = 4 for CTRL and *n* = 4 for ALS patients). (**L**) Quantification of the number of cells with elevated levels of phosphorylated MARK2 in (I) (*n* = 4 for CTRL and *n* = 4 for SOD1^G93A^). (**M**) Quantification of the number of cells with elevated levels of phosphorylated MARK2 in (J) (*n* = 4 for CTRL and *n* = 6 for ALS patients). Scale bars: 50 μm. Error bars represent ± SEM. *p ≤ 0.05; **p ≤ 0.01; ***p ≤ 0.001; ****p ≤ 0.0001. The data underlying the figure can be found in S1 Data. ALS, amyotrophic lateral sclerosis; CTRL, control; eIF2α, eukaryotic initiation factor 2 alpha; MARK2, microtubule affinity-regulating kinase 2; NTg, nontransgenic; PKCδ, protein kinase C delta; Pre, presymptomatic; SOD1, Cu/Zn superoxide dismutase; Symp, symptomatic; WT, wild-type.

Next, we examined the status of the PKCδ-MARK2-eIF2α signaling pathway in ALS mouse models and patients. To address whether PKCδ is activated by misfolded SOD1 proteins, and since the phosphorylation of PKCδ-^505^T is necessary for its kinase activity [40], we analyzed the phosphorylation of PKCδ-^505^T in the spinal cords of transgenic mice expressing various ALS-linked SOD1 mutants. When compared to age-matched nontransgenic and SOD1^WT-YFP^ transgenic mice, SOD1^G85R-YFP^ mice at the symptomatic stage exhibited a substantial increase in the phosphorylation of PKCδ-^505^T, as shown by immunoblotting of spinal cord tissues (Fig 5A). Presymptomatic SOD1^G85R-YFP^ mice did not show such changes in the phosphorylation of PKCδ, whereas in transgenic mice expressing another disease mutant, SOD1^G93A^, similar levels of up-regulation of PKCδ phosphorylation were seen at the symptomatic stage (Fig 5A). As a control, we asked whether phosphorylation of PKCδ at another known site, tyrosine 311 (^311^Y), a docking site unrelated to kinase activity but used for signal-regulated scaffolding [40], is affected by mutant SOD1. In the spinal cords of symptomatic mutant SOD1 transgenic mice, immunoblots showed no change in the levels of phosphorylated PKCδ-^311^Y as compared to SOD1^WT^ or nontransgenic controls (S6A Fig). Furthermore, the changes in the levels of phosphorylated MARK2-^595^T, which indicate MARK2 activity toward eIF2α, were in complete accordance with the activation of PKCδ in the mouse spinal cords (Fig 5A–5C). Additionally, consistent with the previous reports of ER stress in the SOD1 mouse models of ALS [46,47], the phosphorylation of PERK, but not that of GCN2, was increased in the spinal cord tissues from symptomatic mutant SOD1 mice (S6B Fig), suggesting that both PERK and MARK2 could contribute to the phosphorylation of eIF2α in these mice. Together, these results suggest that the PKCδ-MARK2-eIF2α signaling is a previously unrecognized pathway implicated in the neurodegenerative mouse models associated with misfolded SOD1 proteins.

To extend our findings to human patients, we performed immunoblot analysis of PKCδ and MARK2 in spinal cord tissues from ALS patients and non-ALS controls. Of the 13 ALS patients’ spinal cords examined, most showed a substantial increase in the phosphorylation of PKCδ-^505^T when compared to non-ALS controls (Fig 5D and 5E, S7A Fig, and S1 Table), suggesting that activation of PKCδ in the spinal cord is a general feature in ALS patients. In the patients’ tissues that showed up-regulated phosphorylation of PKCδ-^505^T, the phosphorylation of MARK2-^595^T was also significantly increased (Fig 5D and 5F, and S7A Fig). The increases in the phosphorylation of PKCδ and MARK2 were correlated with the increase in the phosphorylation of eIF2α-^51^S in the patients’ spinal cords (Fig 5D and 5G, and S7A Fig). Furthermore, we performed immunohistochemical staining of the spinal cords from ALS patients including one carrying the mutation SOD1^A4V^ using antibodies against phosphorylated PKCδ-^505^T and confirmed that the PKCδ phosphorylation was markedly increased in the affected spinal cords (Fig 5H and 5K). These results demonstrate that the increase in the catalytically active form of PKCδ, as marked by phosphorylated PKCδ-^505^T, is a pathological feature of neurodegeneration in ALS patients.

We also investigated the neuropathology of MARK2 in ALS patients and animal models. In nontransgenic control mice, immunostaining with an antibody specific for MARK2-^595^T showed a diffuse pattern in the gray matter of the anterior horn of the spinal cord. However, in age-matched symptomatic SOD1^G93A^ mice, the staining was markedly increased in both the gray and white matter (Fig 5I and 5L). A subset of neurons had intense staining in the soma. A similar increase in the staining of phosphorylated MARK2-^595^T was observed in the brain cortex, striatum, and midbrain of the SOD1^G93A^ mice (S6C–S6E Fig). Furthermore, we studied the pattern of phosphorylated MARK2 in human spinal cord tissues. In heathy control cases, the immunohistochemical staining of phosphorylated MARK2 was relatively light, except for a set of well-demarcated large neurons. In contrast, in the familial ALS patient with the SOD1^A4V^ mutation, the signals for phosphorylated MARK2 in the cells were increased in both number and intensity (Fig 5J and 5M). Moreover, in a set of 4 sporadic ALS patients that were examined, a similar increase in the staining of phosphorylated MARK2-^595^T was also observed in both the spinal cord and motor cortex (S7B and S7C Fig). These results suggest that the activation of MARK2 is a notable pathologic feature in the spinal cords of ALS patients who suffer from the motor neuron degeneration.

## Discussion

In the present study, we describe a previously unrecognized signaling pathway whereby the proteotoxic stress regulates translation through eIF2α phosphorylation (S8 Fig). We identified MARK2 as a direct kinase for eIF2α in this stress-response signaling. PKCδ acts upstream of MARK2 and senses the protein misfolding stress through its interaction with the molecular chaperone HSP90. The identification of this pathway unveils a distinct stress response mechanism that is important for cytosolic protein homeostasis. Furthermore, the activation of the PKCδ-MARK2-eIF2α pathway in ALS mouse models and human patients associated with protein misfolding suggests that this stress signaling may be important for the pathogenesis of relevant neurodegenerative diseases.

The rate-limiting step in protein synthesis in eukaryotes is at the level of translation initiation [2]. One of the key regulatory factors for translation initiation, eIF2α, is phosphorylated at the conserved residue serine 51 by 4 previously known kinases, including PKR, PERK, HRI, and GCN2, which mediate different stress signals in an integrated stress response network. Although it was previously suggested that there are only 4 eIF2α kinases [48], we provide both in vitro and in vivo evidence, including results from knockout cells lacking the 4 known eIF2α kinases, that MARK2 is a previously unrecognized kinase for eIF2α and that it plays an important role in mediating the phosphorylation of eIF2α upon proteotoxic stress. The 4 previously known eIF2α kinases are closely related phylogenetically and their kinase domains are similar structurally, while MARK2 is phylogenetically distinct [49], thus it would be interesting to investigate the structural basis for MARK2 to recognize eIF2α in future studies. The observation that cells lacking all 5 eIF2α kinases, including MARK2, were still capable of exhibiting a trend for enhanced phosphorylation of eIF2α, albeit to a diminished degree, in response to stress suggests that there may be other factors influencing eIF2α phosphorylation or dephosphorylation. Together, these results expand our understanding of the pathways in the integrated stress response and reveals MARK2 as a distinct signaling hub for the regulation of translation.

In the present study, we also identified PKCδ as an upstream kinase that promotes both basal and induced phosphorylation of eIF2α. PKCδ does not directly phosphorylate eIF2α, but instead acts as a direct kinase of MARK2. It is a multifunctional kinase that influences several cellular processes, including growth, differentiation, and apoptosis [50,51]. The PKCδ-MARK2-eIF2α pathway identified in this study demonstrates a role for PKCδ in the fundamental cellular regulation of translational control. Our identification of a stress-dependent interaction between PKCδ and HSP90 suggests that PKCδ can sense changes in the levels of misfolded proteins through its competitive binding to HSP90. This scenario is reminiscent of the mechanism underlying the activation of HSF1 by unfolded proteins through a dynamic interaction between HSF1 and HSP90 [52], or the activation of PERK by unfolded proteins in the ER lumen through a dynamic interaction between PERK and the ER chaperone protein BiP (immunoglobulin heavy-chain-binding protein) [53]. We have demonstrated that PKCδ-MARK2-eIF2α signaling is activated by protein misfolding stress, independently of PERK. Thus, the identification of the PKCδ-MARK2-eIF2α pathway provides a mechanism for direct signal transduction from cytosolic protein misfolding to translational control.

Most neurodegenerative diseases are associated with toxicities resulting from the accumulation of misfolded proteins, but the molecular and cellular consequences of the protein misfolding stress have not been fully determined. The activation of the PKCδ-MARK2-eIF2α pathway seen in the ALS models and patients’ tissues examined in the present study suggests that translational regulation is one of the pathological consequences of the disease. The translational attenuation as a result of the activated PKCδ-MARK2-eIF2α pathway may first serve as an adaptive stress response that lowers the protein burden during proteotoxic stress. However, a prolonged activation of the pathway under chronic stress could induce built-in mechanisms of cell death [54,55]. In agreement with the importance of translational regulation for neuronal health, increased eIF2α phosphorylation is a common pathological hallmark of major neurodegenerative diseases [56–58]. Moreover, modulation of eIF2α phosphorylation have been shown to affect the phenotypes of animal models of neurodegeneration with different outcomes of alleviation or aggravation of disease phenotypes associated [59–61]. In sum, our results in the present study provide a previously unrealized mechanism for regulating translation during stress and neurodegenerative conditions. Further studies of this signaling pathway may expand our understanding of the regulation of protein homeostasis and its role in the development of relevant human diseases.

## Methods

### DNA constructs

For mammalian expression, human SOD1^WT^ and SOD1^G85R^ were subcloned into the pEF-BOS plasmid as previously described [45]. The pEGFP-C3 expression plasmid was obtained from Addgene (6082–1). The human MARK2 expression plasmid (HsCD00074644) was obtained from the DNASU repository. Both eIF2α and MARK2 were subcloned into the pDEST plasmid using the Gateway system (Thermo Fisher, United States of America). Kinase-dead MARK2^KD^ mutant was generated by PCR, amplifying the fragment without the kinase domain. eIF2α^S51A^ and eIF2α^S51D^ mutants were constructed using the Q5 Site-Directed Mutagenesis Kit (New England Biolab E0554). For the NanoBRET assay, eIF2α^WT^, eIF2α^S51A^, or eIF2α^S51D^ was subcloned into the pHTN HaloTag CMV-neo Vector (Promega JF920304). MARK2 or PERK was subcloned into the pNLF1-C [CMV/Hygro] Vector (Promega KF811458). The HaloTag and NanoLuc expression vectors as well as the positive control of p53 and MDM2 fusion expression vectors are included in the NanoBRET PPI Systems Kit (Promega, USA).

### Genome editing using the CRISPR-Cas9 system

The specific gRNA sequences were selected by using the CRISPR design tool from Benchling. The gRNAs were cloned into the gRNA/Cas9 expression vector pLenti-CRISPR v2, conferring resistance to puromycin (Addgene 52961) or blasticidin (Addgene 98293), or the gRNA multiplexing system STAgR [62]. After cell transduction with the lentiviruses expressing the Cas9/gRNAs, single cell colonies were isolated based on puromycin or blasticidin resistance. The resulting cell lines were verified for their genotypes by sequencing the targeted locus or probing the targeted protein through immunoblot analysis.

### Cell lines

MEFs were grown in Dulbecco’s modified Eagle’s medium (DMEM) supplemented with 10% fetal bovine serum (FBS) and antibiotic–antimycotic solution at 37°C with 5% CO2. The MEFs include knockout lines lacking PERK [63], GCN2 [64], HRI [65], PKCδ [66], MARK2 [35], or PKR [36], and a knockin line eIF2α^S51A^ MEFs [67]. MARK2 stable cell lines were generated by transfecting WT MEF cells with the MARK2^WT^ expression construct (DNASU HsCD00074644) or the MARK2^T595A^ mutant version and then passaged into selective medium containing 3 μg/ml puromycin. Human SOD1^WT^ and SOD1^G85R^ stable cell lines were generated by transfecting WT MEF cells with the CMV.TO-3XnFlag-SOD1^WT^-pkg-tetR-Puro or CMV.TO-3XnFlag-SOD1^G85R^-pkg-tetR-Puro vector and then selecting with 3 μg/ml puromycin. To generate the MEF lines lacking multiple eIF2α kinases, the CRISPR-Cas9 system was used to knock out PEKR, GCN2, HRI, and MARK2 in the existing PKR knockout MEF line [36]. A remnant C-terminal fragment of PKR in the knockout MEF line was further deleted using the CRISPR-Cas9 system. The detection of the C-terminal fragment of PKR was achieved by treating cells with mIFN-α (mouse interferon-α, Biolegend 752804, USA) to induce PKR expression followed by immunoblotting with an antibody against PKR (Santa Cruz SC-6282, USA) [37]. The production of lentiviruses and cell transduction were performed using a previously described protocol with modifications [68]. Single cell colonies that survived puromycin selection were individually expanded in the selective medium to establish independent lines. HSP90 knockdown in MEFs was achieved by infecting cells with virus derived from pLenti-CRISPR v2 harboring the HSP90-specific gRNA (5′-ACCCCAGTAAACTGGACTCG-3′), and a population of puromycin-selected cells were used. Human MARK2 knockout cells were generated using CRISPR-Cas9 editing in a haploid human HAP1 cell line (HZGHC000328c013) (Horizon Discovery, United Kingdom). HAP1 cell lines were cultured in Iscove’s modified Dulbecco’s medium (IMDM) with 10% FBS. HEK293 cells were grown in DMEM with 10% FBS.

### Immunoblotting

Cells were washed twice with 1 X PBS and then lysed and harvested on ice in RIPA solution (50 mM Tris-HCl (pH 7.6); 150 mM NaCl; 1% NP-40; 1% SDS; 100 mM sodium fluoride; 17.5 mM β-glycerophosphate; 0.5% sodium deoxycholate; 10% glycerol). The RIPA buffer was supplemented with EDTA-free protease inhibitor cocktail (Roche, USA), phosphatase inhibitor cocktail 2 and phosphatase inhibitor cocktail 3 (Sigma-Aldrich, USA), 1 μM phenylmethanesulfonyl fluoride, and 2 μM sodium orthovanadate. Lysates were kept cold on ice, pulse-sonicated for 10 min, and then centrifuged at 12,000*g* at 4°C for 10 min. The protein content of each sample was determined by a bicinchoninic acid (BCA) assay (Thermo Fisher). Equal amounts of total protein extract were resolved by SDS-PAGE and transferred to nitrocellulose membranes (Millipore HATF08550, USA). The blots were blocked with 5% w/v BSA and 0.05% NaN_3_ in TBST and incubated with primary antibodies at 4°C overnight, then finally incubated with appropriate secondary antibodies. The antibodies used include those against eIF2α (Cell Signaling 5324, USA), peIF2α (Cell Signaling 9721), PERK (Cell Signaling 3192), pPERK-^980^T (Cell Signaling 3179), PKR (Santa Cruz SC-708 and SC-6282), HRI (Millipore 07–728), GCN2 (Cell Signaling 3302), pGCN2-^899^T (Abcam ab75836, USA), ATF4 (Cell Signaling 11815), HSP90 (Cell Signaling 8165), HSP70 (Cell Signaling 4872), Flag (Sigma F1804), Actin (Santa Cruz SC-47778), Tubulin (Proteintech 10068-1-AP, USA), PKCδ (Santa Cruz SC-937; Cell Signaling 2058), pPKCδ-^311^Y (Cell Signaling 2055), pPKCδ-^505^T (Cell Signaling 9374), MARK2 (Abcam ab135816; Santa Cruz SC-365405), pMARK2-^595^T (Abcam ab34751), PP1α (Cell Signaling 2582), pPP1α-^320^T (Cell Signaling 2581), and puromycin (Millipore MABE343). Images were captured with an Odyssey imager and analyzed with Image Studio software (Licor 9120, USA).

### In vivo labeling for protein synthesis analysis

Stable MEF cell lines overexpressing MARK2^WT^ or MARK2^T595A^ were plated onto 6-well plates (2 × 10^5^ cells per well) overnight. For puromycin labeling, cells were treated with 10 ug/ml puromycin in culture medium for 10 min and then washed 3 times with 1 X PBS and lysed with RIPA buffer as described above. The cell lysate was analyzed by immunoblotting against puromycin. For ^35^S labeling, cells were incubated with methionine- and cysteine-free DMEM supplemented with 10% FBS (MilliporeSigma F0392) for 1 h. A total of 200 μCi of [^35^S]-methionine and [^35^S]-cysteine (PerkinElmer NEG772002MC, USA) was then added to each dish to metabolically label the cells for 1 h. After radiolabeling, the cells were washed 3 times with 1 X PBS and lysed with RIPA buffer as described above. A total of 20 ng of cell lysate was added into 4 ml of liquid scintillation cocktail (MP Biomedicals 01882475-CF, USA), and the radioactivity was detected using LS6500 Liquid Scintillation Counter (Beckman Coulter, USA).

### Immunoprecipitation

Cells were washed twice with 1 X PBS and then lysed on ice in lysis buffer (50 mM Tris-HCl (pH 7.5); 150 mM NaCl; 1% NP-40; 1 mM EDTA; 0.5% sodium deoxycholate). The lysis buffer was supplemented with EDTA-free protease inhibitor cocktail (Roche). The cell lysates were immunoprecipitated with anti-PKCδ antibody (Cell Signaling 2058) using protein A/G magnetic beads. The beads were washed 3 times with washing buffer (50 mM Tris-HCl (pH 7.5); 150 mM NaCl; 1% NP-40; 1 mM EDTA) and then eluted with low-pH elution buffer at room temperature for 10 min. The eluents were neutralized with 1 M Tris-HCl (pH 8.0) and separated by SDS-PAGE and immunoblotted with antibodies against heat shock proteins including HSP90. For the coimmunoprecipitation analyses of heat shock proteins, MEF cells were heat-shocked at 44°C as previously described [52]. Aliquots of the whole-cell lysates were immunoblotted using Actin and PKCδ antibodies.

### Protein purification

The protein kinases and substrates used in the in vitro kinase activity assays were expressed from bacterial, insect, or mammalian cells. Proteins purified using the *E*. *coli* strain Rosetta include MARK2^WT^, MARK2^T595A^, eIF2α^WT^, and eIF2α^S51A^. The cDNAs encoding these proteins were cloned into the pET28a plasmid with His tags, and the protein expression was induced by IPTG. *E*. *coli* cells were grown until the OD600 reached 0.4 to 0.6 before induction with 0.1 mM IPTG at 16°C for 24 h. *E*. *coli* cells were harvested and suspended using lysis buffer (50 mM NaH_2_PO_4_; 300 mM NaCl; 10 mM imidazole; 0.05% Tween 20 (pH 8.0); and EDTA-free protease inhibitor cocktail [Roche]). Cells were kept cold on ice, lysed with a French pressure cell for 10 to 15 min, and then centrifuged at 10,000*g* at 4°C for 30 min. The lysates were immunoprecipitated using Ni-NTA agarose (Qiagen 30210, USA) at 4°C for 1 h. The Ni-NTA agarose was washed with washing buffer (50 mM NaH_2_PO_4_; 300 mM NaCl; 20 mM imidazole; 0.05% Tween 20 (pH 8.0)) twice and the protein eluted by using elution buffer (50 mM NaH_2_PO_4_; 300 mM NaCl; 250 mM imidazole; 0.05% Tween 20 (pH 8.0)). The eluted proteins were passed through molecular weight cut-off centrifugal filters (Millipore) to remove imidazole and stored in buffer (20 mM Tris-HCl; 150 mM NaCl; 0.1 mM DTT) at −80°C. In addition, recombinant proteins expressed in Sf9 insect cells after infection with recombinant baculovirus include GST-tagged PKR, PKCδ, TTK, BMPR1A, and MARK2 and His-tagged PYK2 and eIF2α. These proteins were purified using a standard protocol with affinity column chromatography on glutathione columns by SignalChem (Canada). The purified proteins were diluted in a kinase buffer with 0.05 nM DTT. MBP proteins that were used as the universal kinase substrate were obtained from SignalChem (M42-51N). For mammalian expression of recombinant proteins, MARK2^WT^, MARK2^T595A^, and a GFP control were expressed and purified from HEK293 cells. The cDNAs were cloned into a modified pCDNA3.1 plasmid to express Flag-tagged proteins in a tet-inducible manner as described previously [45]. The constructs were transfected into HEK293 cells, which were treated with 0.5 ug/ml doxycycline to induce expression. The cells were lysed in RIPA buffer (50 mM Tris-HCl; 150 mM NaCl; 1% NP-40; 1 mM PMSF (pH 8.0); and EDTA-free protease inhibitor cocktail [Roche]). The cell lysates were incubated with anti-Flag M2 magnetic beads (SIGMA M8823) for 24 h at 4°C. The beads were then washed with washing buffer (50 mM Tris-HCl (pH 7.5); 150 mM NaCl) several times. The proteins were eluted from the beads by adding 5 volumes of 5 μg/μl 3xFlag peptide solution, followed by incubation at 4°C for 1 h.

### In vitro kinase activity and kinetics assays

For the in vitro kinase activity assay based on radiolabeling and gel electrophoresis, the reaction mix included a kinase protein at 0.04 μg/μl (MARK2 and MARK2^T595A^ at 0.51 μM, PKR at 0.54 μM, PKCδ at 0.51 μM, and the control GFP at 1.48 μM) and a substrate protein at 0.2 μg/μl (MBP at 9.3 μM, eIF2α at 5.26 μM, and MARK2 at 2.56 μM), 50 μM cold ATP, and [γ-^32^P]-ATP (1 mCi/100 μl, PerkinElmer) diluted 1:300 in the kinase assay buffer (SignalChem, K01-09). The reactions were incubated at 30°C for 15 min before being analyzed by SDS-PAGE. Radioactive signals were detected with a FLA7000 imager (Fujifilm FLA7000, USA). For the in vitro kinase kinetic analysis, the Kinase-Glo assay was used to measure kinase activities by quantifying ATP consumption via luminescent signals (Promega V6711). In the initial round of analysis, the kinase proteins were serially diluted as indicated, while the substrate protein MBP was kept constant at 0.1 μg/μl (4.65 μM) with 5 μM of ATP supplemented. In the subsequent round of analysis, the Km concentrations of PKR and MARK2 as determined above were used, while eIF2α as the substrate was serially diluted as indicated, with 5 μM of ATP supplemented. The MARK^KD^ mutant was used at the same concentrations as those of its WT counterpart. The reactions were incubated at 30°C for 60 min before addition of 1:1 volume of the Kinase-Glo reagent (Promega), followed by incubation at room temperature for 10 min. The Luminescence was detected with the Synergy H1 microplate reader (BioTek, USA).

### NanoLuc-based bioluminescence resonance energy transfer assays

The NanoBRET assays were performed according to the manufacturer’s protocol (Promega NanoBRET Protein:Protein Interaction System), with some modifications. For each individual population, cells were seeded at 2 × 10^5^ cells/ml into 96-well plates (Corning Costar 3917 white opaque assay plates) and incubated in DMEM supplemented with 10% FBS and antibiotic–antimycotic solution at 37°C with 5% CO_2_ for 24 h. After 24 h, the cells were cotransfected with a combination of a NanoLuc fusion protein vector and a HaloTag fusion protein vector using jetPRIME Transfection Reagent and incubated at 37°C, 5% CO_2_ for 16 to 24 h. After 24 h, NanoBRET Nano-Glo Substrate (Promega) was added to the transfected cells, and the fluorescence signal was measured at 460 nm and 618 nm within 10 min of substrate addition. A Synergy H1 Hybrid Reader (BioTek) with a custom filter cube (450 nm / 610 nm) was used to measure the luminescence values (6 mm read height, 1 s integration time, 100 to 160 gain value). Mean corrected milliBRET (mBU) values were calculated using equations from the manufacturer’s protocol (Promega, NanoBRET PPI Systems, N1821).

### Mouse and human tissues

The SOD1 transgenic mice used in this study have been previously characterized: the SOD1^G93A^ line [B6SJL-TgN (SOD1^G93A^)1Gur; Jackson Laboratory] [69] and the SOD1^G85R-YFP^ and SOD1^WT-YFP^ lines [26]. Transgenic mice were identified by PCR amplification of DNA extracted from tail biopsies. Mice were euthanized in a CO_2_ chamber, and fresh tissues were harvested by flash-freezing in liquid nitrogen and then stored at −80°C. For immunoblot analysis, spinal cords were rinsed with cold PBS and homogenized with cold RIPA buffer using glass tissue grinders. The homogenates were then centrifuged at 4°C at 1,000*g* for 10 min, and the supernatants were centrifuged again at 16,000*g* for 10 min, with the final supernatant used for immunoblot analysis. The animal protocol (MO18H105) was approved by the Animal Care and Use Committee of the Johns Hopkins Medical Institutions. Human postmortem brain and spinal cord tissues used in this study are deidentified by independent sources and described in S1 Table.

### Immunofluorescent staining and immunohistochemistry

For immunofluorescent staining, mouse tissues were fixed in 4% paraformaldehyde and then sectioned at 20 μm on a cryostat. Slices were rinsed 3 times with PBS and treated with blocking solution (5% normal goat serum, 0.1% Tween 20 in 1X TBS) for 1 h at room temperature. Slices were incubated with a primary antibody (peIF2α, Cell Signaling; pPKCδ-^505^T, Cell Signaling; pMARK2-^595^T, Abcam) at 4°C overnight. Then the slices were washed 3 times with PBS and incubated with a fluorochrome-conjugated secondary antibody (anti-rabbit, Alexa Fluor 594; Invitrogen, USA, 1:400) for 2 h at room temperature. After 3 to 5 times of washes with PBS, the slices were coverslipped in mounting medium containing DAPI.

The ALS patient samples were fixed with 4% PFA prior to paraffin embedding. Paraffin-embedded tissue blocks were sectioned at 10 μm using a microtome. Tissue sections were mounted on Superfrost Plus slides, left to dry at room temperature for 24 h, and stored in −80°C. For use, the sections were heated at 65°C for 30 min, cleared with xylene, deparaffinized, and hydrated through a series of graded anhydrous, histological grade ethanol solutions, then washed 3 times with xylenes and 100% EtOH, one time with the graded EtOH series of decreasing concentrations, and twice with deionized water. The sections were then rinsed with TBS and underwent antigen unmasking by incubating the slides in sub-boiling 10 mM citrate buffer (pH 6.0) for 10 min. Sections were cooled to room temperature and then underwent three 5-min washes with TBS. Endogenous peroxidase activity was quenched using a 10% methanol and 3% H_2_O_2_ solution in TBS for 10 min at room temperature. Afterwards, sections were washed twice with TBS and incubated with blocking buffer solution (0.3% Triton-X 100, 5% normal goat serum, 1% BSA in TBS) inside a humidified chamber for 30 min at room temperature. Blocking solution was aspirated, and sections were incubated with a primary antibody diluted in the blocking buffer solution (MARK2; 1:400) overnight at 4°C. The next day, after equilibrating to room temperature, sections were washed with TBST and treated with a micropolymerized peroxidase reporter, ImmPRESS Reagent Anti-Rabbit IgG (Vector Laboratories, USA), in a humidified chamber for 30 min at room temperature. The tissues were rinsed with TBST, and the peroxidase reporter was detected using the ImmPACT DAB (3,3′-diaminobenzidine tetrahydrochloride) peroxidase substrate (Vector Laboratories). Tissue sections were incubated in the substrate working solution at room temperature until suitable staining developed, which was approximately 3 min. Slides were then rinsed again 3 times for 10 min in TBS and underwent dehydration with a series of graduated alcohols. Finally, the sections were cleared with three 5-min incubations in xylene and coverslipped with VectaMount Permanent Mounting Medium (Vector Laboratories).

### Microscopy

Mouse tissue immunofluorescent staining was viewed with a Leica SP8 confocal fluorescence microscope. Z-stack images were taken and processed into a maximal projected image. Human samples stained by immunohistochemistry were viewed using brightfield microscopy on a Nikon Eclipse Ti-S microscope equipped with a high-definition color camera head, DS-Fi2, and DS-U3 control unit. Images were taken and assessed with NIS Elements Documentation Imaging Software (Nikon, USA) and analyzed using ImageJ software.

### Statistical analysis

The statistical analyses were performed with Student t tests for 2-group comparisons and one-way ANOVA with the Tukey post hoc test for multiple group comparisons using Graphpad Prism software. The sample size “n” represents independent experiments unless otherwise indicated. *P* values less than 0.05 were considered significant.

## Supporting information

S1 Fig MARK2 is a specific and direct kinase for eIF2α. (**A**) Coomassie blue gel staining confirms the high purity of the proteins used in the in vitro kinase activity assays. (**B**) In vitro kinase assays using purified proteins and [γ-^32^P]-ATP demonstrate that PKCδ is not a direct kinase for eIF2α. MBP was used as a positive control substrate for the kinase activity of PKCδ. PKR was used as a positive control for eIF2α kinase activity (lane 5). (**C–E**) Kinetic analysis of the reactions between the kinase, PKR, MARK2^WT^, or MARK2^KD^ (kinase-dead mutant), and the substrate MBP using the Kinase-Glo assay quantifying ATP consumption via luminescent signals. Initial velocities represented by ATPs incorporated into the substrate were plotted against the kinase to determine the Km and Vmax of PKR, MARK2^WT^, and MARK2^KD^. (**F–H**) Kinetic analysis of the reactions between the kinases and the substrates eIF2α^WT^ and eIF2α^S51A^ using the Kinase-Glo assay. (**I**) In vitro kinase assays based on radiolabeling and gel electrophoresis using proteins purified from *E*. *coli* demonstrate that MARK2 directly phosphorylates eIF2α at serine 51. The kinase-dead MARK2^KD^ mutant did not show activity toward eIF2α^WT^ or eIF2α^S51A^. The data underlying the figure can be found in S1 Data. eIF2α, eukaryotic initiation factor 2 alpha; MARK2, microtubule affinity-regulating kinase 2; MBP, myelin basic protein; PKCδ, protein kinase C delta; PKR, protein kinase R; WT, wild-type.
(TIF)

S2 Fig Schematics of CRISPR editing and NanoBRET analysis. The CRISPR/Cas9-induced null mutations were generated to create knockout cells lacking single or multiple eIF2α kinases. The 4-KO MEFs were generated by deleting PERK, HRI, and GCN2 from an existing PRK knockout MEF line. The 5-KO MEFs were generated by deleting MARK2 from the 4-KO MEFs lacking PERK, PRK, HRI, and GCN2. The 4-KO′ and 5-KO′ MEFs were generated by introducing deletion mutations in exon 5 of the PKR gene, resulting in the removal of a remnant C-terminal fragment of PKR from the existing 4-KO and 5-KO MEF lines. In addition to Sanger sequencing to confirm the DNA mutation, the deletion of PERK, PKR, HRI, GCN2, and MARK2 was verified by immunoblotting. (**A**) In the MARK2 knockout HAP1 cell line, the human MARK2 gene is disrupted with a CRISPR/Cas9-induced 11-bp deletion (GATTCGGGGCC) in exon 2, resulting in a premature stop codon (TGA) in exon 2 and disruption of the MARK2 gene in the near-haploid genome. (**B**) In the 5-KO MEF line, the MARK2 gene is disrupted with a CRISPR/Cas9-induced 1-bp insertion in exon 2, resulting in a premature stop codon (TGA) in exon 2 in both alleles of the gene. (**C**) In the 4-KO and 5-KO MEF lines, the PERK gene is disrupted with a CRISPR/Cas9-induced 1/2-bp deletion in exon 1, resulting in a premature stop codon (TGA or TAA) in exon 2 in both alleles of the gene. (**D**) In the 4-KO and 5-KO MEF lines, the HRI gene is disrupted with a CRISPR/Cas9-induced 1-bp or 4-bp deletion in exon 1, resulting in a premature stop codon (TAA) in exon 2 in both alleles of the gene. (**E**) In the 4-KO and 5-KO MEF lines, the GCN2 gene is disrupted with a CRISPR/Cas9-induced 13-bp or 6-bp deletion in exon 2, resulting in a premature stop codon (TGA or TAA) in exon 2 in both alleles of the gene. (**F**) In the 4-KO′ and 5-KO′ MEF lines, the existing PKR knockout allele (4-KO and 5-KO) is further edited using CRISPR to disrupt a remnant C-terminal fragment of PKR. In the original PKR knockout allele, exons 2 and 3 were replaced with a segment containing the NEO-UMS cassette, which functions as a translational stop. Due to exon skipping, a C-terminal fragment of PKR containing dsRBM2 and the kinase domain could still be generated as depicted. To remove the C-terminal fragment, we used CRISPR to introduce a 1-bp or 11-bp deletion in exon 5 at the 2 alleles, resulting in premature stop codons (TAA or TAG) in exon 5 or 6, respectively, of the 2 alleles. (**G**) Workflow for the NanoBRET assay to monitor the interaction between MARK2 and eIF2α in live HEK293 cells. eIF2α, eukaryotic initiation factor 2 alpha; GCN2, general control nonderepressible factor 2 kinase; HRI, heme-regulated eIF2α kinase; MARK2, microtubule affinity-regulating kinase 2; MEF, mouse embryonic fibroblast; NEO-UMS, neomycin and upstream mouse sequence; PERK, PKR-like ER-resident kinase; PKR, protein kinase R.
(TIF)

S3 Fig The activation of MARK2 is correlated with the phosphorylation of eIF2α, and the signaling pathway is independent of the previously known kinases. (**A**) Deletion of the MARK2 gene decreases the phosphorylation of eIF2α-^51^S in human HAP1 cells. Bar graph represents quantification of the immunoblots (*n* = 3). (**B**) Immunoblotting analyses of MEFs treated with the proteasome inhibitor MG132 (500 nM) indicate increased levels of phosphorylated eIF2α-^51^S that correlated with the levels of phosphorylated MARK2-^595^T over the 24-h time course of the MG132 treatment. The graph used to calculate the Pearson coefficient is shown to indicate the significant correlation (*p* = 0.0161). (**C-F**) Immunoblotting analyses of WT and knockout MEFs treated with MG132 indicate that the PKCδ-MARK2-eIF2α signaling pathway can be activated, as measured by the levels of phosphorylated eIF2α-^51^S, MARK2-^595^T, and PKCδ-^505^T independently of any of the previously known eIF2α kinases, including PERK, HRI, PKR, and GCN2. Bar graphs represent the quantification of the immunoblots (*n* = 3). Error bars represent ± SEM. *p ≤ 0.05; **p ≤ 0.01. The data underlying the figure can be found in S1 Data. eIF2α, eukaryotic initiation factor 2 alpha; GCN2, general control nonderepressible factor 2 kinase; HRI, heme-regulated eIF2α kinase; KO, knockout; MARK2, microtubule affinity-regulating kinase 2; MEF, mouse embryonic fibroblast; PERK, PKR-like ER-resident kinase; PKCδ, protein kinase C delta; PKR, protein kinase R; WT, wild-type.
(TIF)

S4 Fig MARK2-mediated eIF2α-^51^S phosphorylation, translational attenuation, and the characterization of MEFs lacking multiple eIF2α kinases. (**A**) The specificity of the antibody against phosphorylated eIF2α-^51^S was verified in an eIF2α^S51A^ knockin mutant MEF line, in which the S51A mutation abolished the immunoblot signal of phosphorylated eIF2α-^51^S observed in WT MEFs treated with MG132. (**B**) MEFs stably overexpressing MARK2^WT^ or MARK2^T595A^ were pulsed-labeled with puromycin for 10 min, and the cell lysates were analyzed by SDS-PAGE and immunoblotting against puromycin-labeled proteins. The bar graph represents the quantification of the immunoblots (*n* = 3). (**C**) MEFs stably overexpressing MARK2^WT^ or MARK2^T595A^ were pulse-labeled with ^35^S-methionine and ^35^S-cysteine for 1 h, and the cell lysates were analyzed by liquid scintillation counting for ^35^S-labeled proteins (*n* = 3). The quantitative results indicate that overexpression of MARK2^WT^ caused attenuation of global translation, while the T595A mutation impaired its regulatory activity. (**D**) A time course of the treatment with MG132 (20 μM) at indicated times shows that the phosphorylation of eIF2α-^51^S peaked around 4 h in the MEFs. (**E**) Immunoblot analyses of MEFs treated with sodium arsenite (200 μM, 1 h) indicate that the phosphorylation of MARK2-^595^T and eIF2α-^51^S was increased by the stress in WT and 4-KO cells. Bar graphs represent the quantification of the immunoblots (*n* = 3). (**F**) Immunoblot analyses of MEFs treated with tunicamycin (24 μg/ml, 2 h) indicate that the phosphorylation of MARK2-^595^T was not affected by the stress, while the phosphorylation of eIF2α-^51^S could be independently induced in WT but not the 4-KO cells. Bar graphs represent the quantification of the immunoblots (*n* = 3). (**G**) Immunoblot analyses of WT, 5-KO, 4-KO′, and 5-KO′ MEFs treated with mINF-α (1,000 U/ml for 18 h) indicate a remnant C-terminal fragment of PKR in 5-KO cells, which has been deleted in 4-KO′ and 5-KO′ cells as designed. (**H**) Immunoblot analyses of WT, 4-KO′, and 5- KO′ MEFs treated with MG132 indicate that eIF2α-^51^S is phosphorylated in response to the stress in the 4-KO′ MEFs. The levels of phosphorylated eIF2α-^51^S in the 5- KO′ cells are significantly lower than those in the 4-KO′ MEFs (*n* = 3). The levels of phosphorylated PP1α-^320^T in the 4-KO′ and 5-KO′ cells are significantly lower than those in WT MEFs, and there is a trend for an increase in the phosphorylation of PP1α-^320^T in the 5-KO′ MEFs compared to the 4-KO′ MEFs (*n* = 3; 4-KO′ with MG132 vs. 5-KO′ with MG132, Student *t* test *p* = 0.0191, nonsignificant using one-way ANOVA with the Tukey post hoc test). Error bars represent ± SEM. *p ≤ 0.05; **p ≤ 0.01; ***p ≤ 0.001; ****p ≤ 0.0001. The data underlying the figure can be found in S1 Data. eIF2α, eukaryotic initiation factor 2 alpha; KO, knockout; MARK2, microtubule affinity-regulating kinase 2; MEF, mouse embryonic fibroblast; mINF-α, mouse interferon-α; PKR, protein kinase R; WT, wild-type.
(TIF)

S5 Fig PKCδ interacts with HSP90 but not HSP70 and mediates proteotoxicity-induced activation of PKCδ-MARK2-eIF2α signaling. (**A**) In coimmunoprecipitation analyses, no HSP70 was detected in immunoprecipitates pulled down by the anti-PKCδ antibody from WT MEFs, those from PKCδ KO MEFs, or those from MEFs stably expressing PKCδ. IgG was used as a control for the anti-PKCδ antibody. (**B**) Comparison of WT MEFs and those with HSP90 knockdown by CRISPR in immunoblot analyses indicate that the down-regulation of HSP90 substantially increased the phosphorylation of PKCδ-^505^T, MARK2-^595^T, and eIF2α-^51^S. eIF2α, eukaryotic initiation factor 2 alpha; HSP70, heat shock protein 70; HSP90, heat shock protein 90; IgG, immunoglobulin G; IB, immunoblotting; IP, immunoprecipitation; KO, knockout; MARK2, microtubule affinity-regulating kinase 2; MEF, mouse embryonic fibroblast; PKCδ, protein kinase C delta; WT, wild-type.
(TIF)

S6 Fig Increased phosphorylation of MARK2 occurs in the affected tissues of a mutant SOD1-induced ALS mouse model. (**A**) Immunoblot analyses of spinal cord lysates from NTg, SOD1^WT-YFP^, presymptomatic and symptomatic SOD1^G85R-YFP^, and SOD1^G93A^ transgenic mice show no change in the levels of phosphorylation of PKCδ at tyrosine 311. (**B**) Immunoblot analyses of spinal cords from symptomatic SOD1^G93A^ mice and NTg littermate controls indicate that the level of phosphorylated PERK-^980^T was significantly increased in the SOD1^G93A^ mice, while no change was detected for GCN2. Immunohistochemical analyses of phosphorylated MARK2 in the brain cortex (**C**), midbrain (**D**), and striatum (**E**) from symptomatic SOD1^G93A^ transgenic mice and NTg controls. The staining of phosphorylated MARK2-^595^T in all 3 brain regions is increased in the SOD1^G93A^ mice as compared to NTg mice. Error bars represent ± SEM. *p ≤ 0.05. Scale bar: 50 μm. The data underlying the figure can be found in S1 Data. ALS, amyotrophic lateral sclerosis; GCN2, general control nonderepressible factor 2 kinase; MARK2, microtubule affinity-regulating kinase 2; n.s., nonsignificant; NTg, nontransgenic; PERK, PKR-like ER-resident kinase; PKCδ, protein kinase C delta; Pre, presympomatic; SOD1, Cu/Zn superoxide dismutase; Symp, symptomatic; WT, wild-type.
(TIF)

S7 Fig ALS patients’ tissues exhibit increased phosphorylation of MARK2. (**A**) Representative immunoblot analyses of PKCδ, MARK2-^595^T, and eIF2α in the spinal cord tissues from ALS patients and non-ALS controls, indicating that increased phosphorylation of PKCδ-^505^T, MARK2-^595^T, and eIF2α-^51^S is a general phenotype in patient tissues. (**B**) Immunohistochemical staining of phosphorylated MARK2-^595^T in the spinal cords from an SOD1^A4V^-ALS patient, an sALS patient, and a non-ALS age-matched control case. (**C**) Immunostaining for phosphorylated MARK2-^595^T in the motor cortex of 3 different sporadic ALS patients. Increased phosphorylation of MARK2-^595^T is observed in all ALS patient tissues. Scale bar: 50 μm. ALS, amyotrophic lateral sclerosis; CTRL, control; eIF2α, eukaryotic initiation factor 2 alpha; fALS, familial ALS; MARK2, microtubule affinity-regulating kinase 2; PKCδ, protein kinase C delta; sALS, sporadic ALS; SOD1, Cu/Zn superoxide dismutase.
(TIF)

S8 Fig The model for the PKCδ-MARK2-eIF2α signaling pathway. Upon protein misfolding stress, HSP90 is sequestered by misfolded proteins, resulting in phosphorylation and activation of PKCδ, which in turn activates MARK2 that phosphorylates eIF2α. The increased phosphorylation of eIF2α leads to translational attenuation. eIF2α, eukaryotic initiation factor 2 alpha; HSP90, heat shock protein 90; MARK2, microtubule affinity-regulating kinase 2; PKCδ, protein kinase C delta.
(TIF)

S1 Table The list of patients’ tissues. (TIF)

S1 Data Numerical data used in figure preparation. (XLSX)

S1 Raw Images Original gel and blot images. (PDF)

## Abbreviations

ALS: amyotrophic lateral sclerosis
ATF4: activating transcription factor 4
BiP: immunoglobulin heavy-chain-binding protein
BMPR1A: bone morphogenetic protein receptor type 1A
BRET: bioluminescence resonance energy transfer
eIF2α: eukaryotic initiation factor 2 alpha
ER: endoplasmic reticulum
FTD: frontotemporal dementia
GCN2: general control nonderepressible factor 2 kinase
HRI: heme-regulated eIF2α kinase
HSP90: heat shock protein 90
IgG: immunoglobulin G
MARK2: microtubule affinity-regulating kinase 2
MBP: myelin basic protein
MEF: mouse embryonic fibroblast
PERK: PKR-like ER-resident kinase
PKCδ: protein kinase C delta
PKR: protein kinase R
PP1: protein phosphatase 1
PYK2: protein tyrosine kinase 2 beta
SOD1: Cu/Zn superoxide dismutase
TTK: TTK protein kinase
WT: wild-type
