# Supplementary figures and images for: MARK2 phosphorylates eIF2α in response to proteotoxic stress
Source: PLoS Biol. 2021 Mar 11;19(3):e3001096. doi: 10.1371/journal.pbio.3001096 (PMC7951919; doi:10.1371/journal.pbio.3001096)

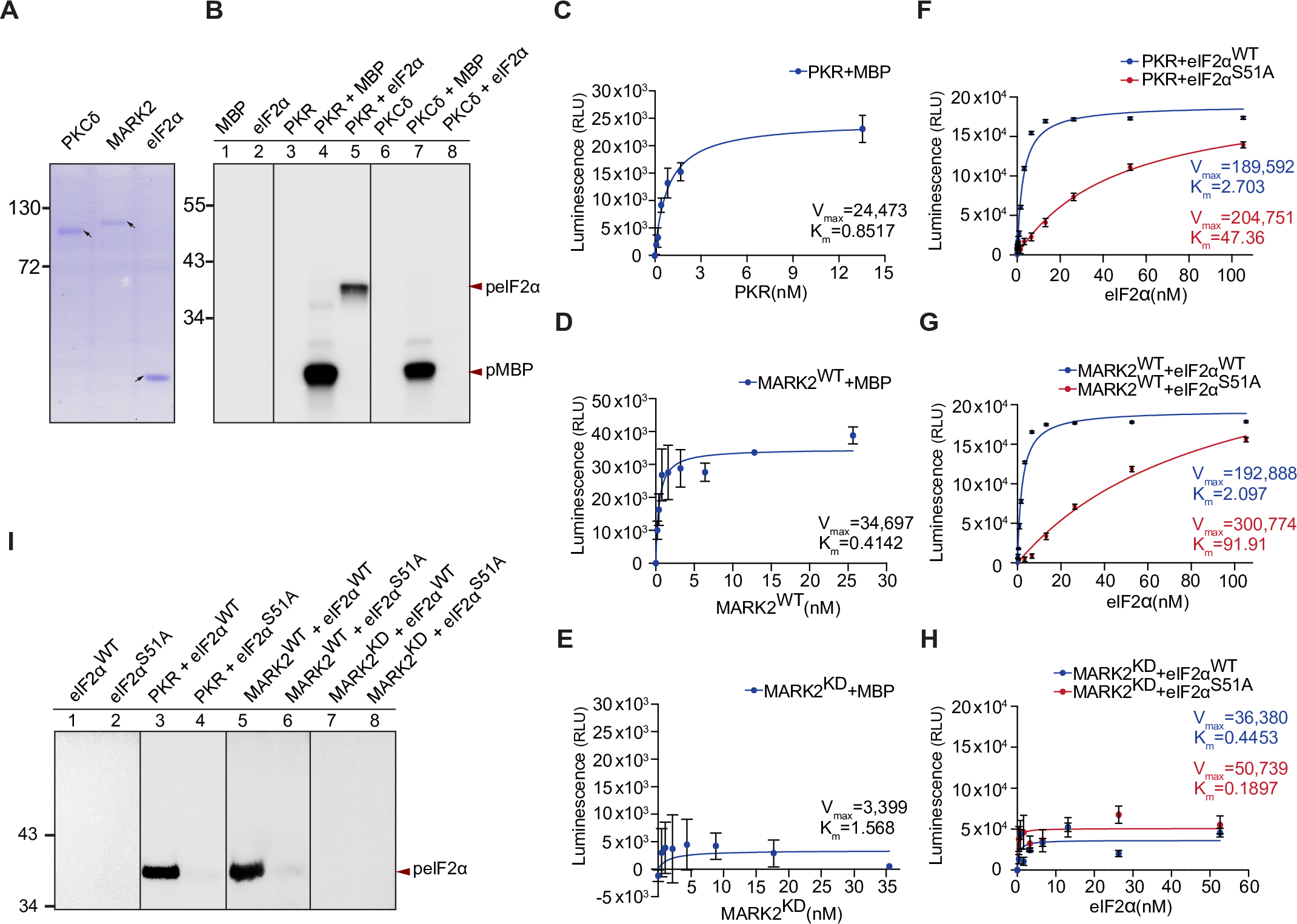

Supplement: S1 Fig — (A) Coomassie blue gel staining confirms the high purity of the proteins used in the in vitro kinase activity assays. (B) In vitro kinase assays using purified proteins and [γ-32P]-ATP demonstrate that PKCδ is not a direct kinase for eIF2α. MBP was used as a positive control substrate for the kinase activity of PKCδ. PKR was used as a positive control for eIF2α kinase activity (lane 5). (C–E) Kinetic analysis of the reactions between the kinase, PKR, MARK2WT, or MARK2KD (kinase-dead mutant), and the substrate MBP using the Kinase-Glo assay quantifying ATP consumption via luminescent signals. Initial velocities represented by ATPs incorporated into the substrate were plotted against the kinase to determine the Km and Vmax of PKR, MARK2WT, and MARK2KD. (F–H) Kinetic analysis of the reactions between the kinases and the substrates eIF2αWT and eIF2αS51A using the Kinase-Glo assay. (I) In vitro kinase assays based on radiolabeling and gel electrophoresis using proteins purified from E. coli demonstrate that MARK2 directly phosphorylates eIF2α at serine 51. The kinase-dead MARK2KD mutant did not show activity toward eIF2αWT or eIF2αS51A. The data underlying the figure can be found in S1 Data. eIF2α, eukaryotic initiation factor 2 alpha; MARK2, microtubule affinity-regulating kinase 2; MBP, myelin basic protein; PKCδ, protein kinase C delta; PKR, protein kinase R; WT, wild-type. (TIF) [file pbio.3001096.s001.tif]

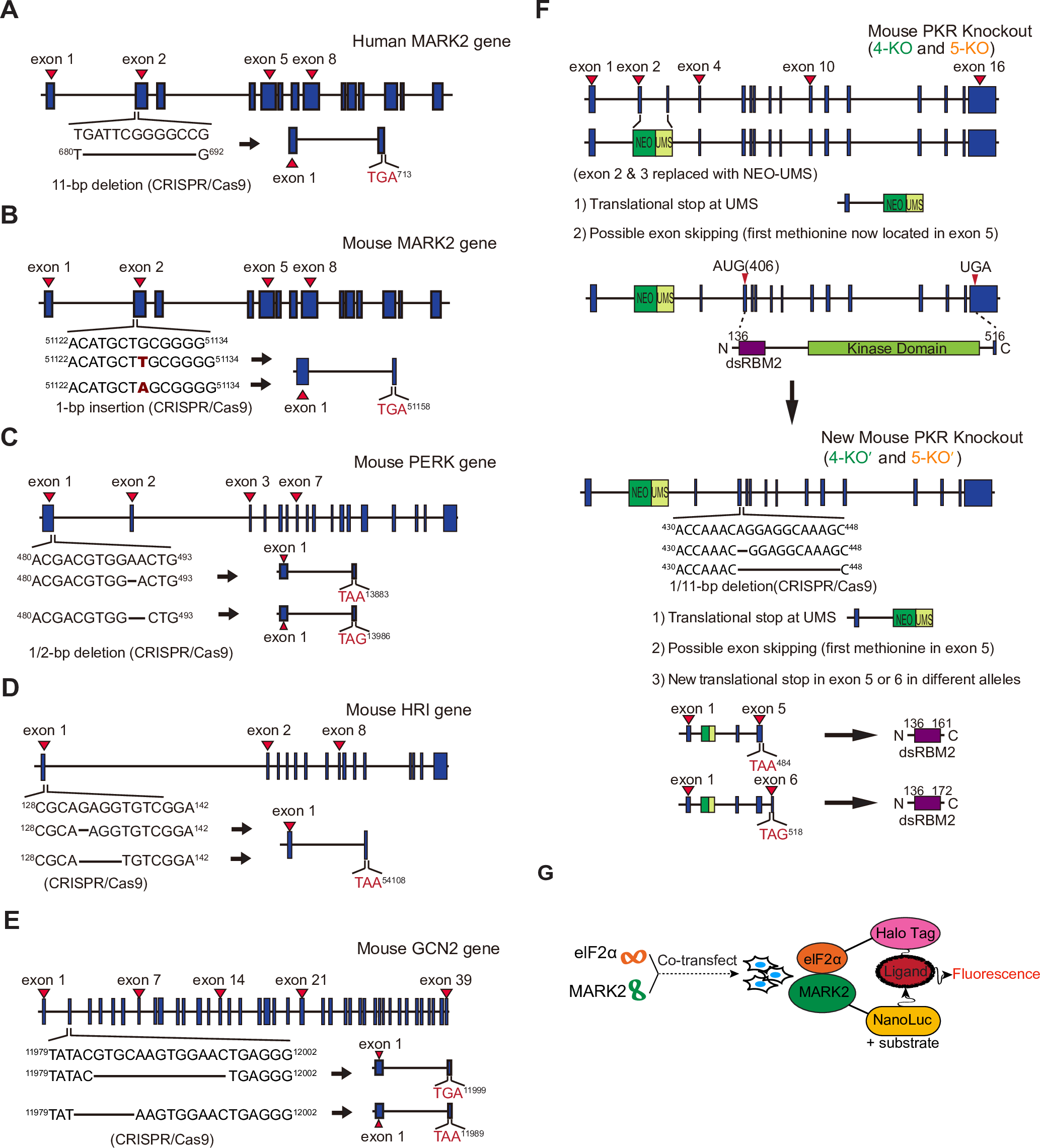

Supplement: S2 Fig — The CRISPR/Cas9-induced null mutations were generated to create knockout cells lacking single or multiple eIF2α kinases. The 4-KO MEFs were generated by deleting PERK, HRI, and GCN2 from an existing PRK knockout MEF line. The 5-KO MEFs were generated by deleting MARK2 from the 4-KO MEFs lacking PERK, PRK, HRI, and GCN2. The 4-KO′ and 5-KO′ MEFs were generated by introducing deletion mutations in exon 5 of the PKR gene, resulting in the removal of a remnant C-terminal fragment of PKR from the existing 4-KO and 5-KO MEF lines. In addition to Sanger sequencing to confirm the DNA mutation, the deletion of PERK, PKR, HRI, GCN2, and MARK2 was verified by immunoblotting. (A) In the MARK2 knockout HAP1 cell line, the human MARK2 gene is disrupted with a CRISPR/Cas9-induced 11-bp deletion (GATTCGGGGCC) in exon 2, resulting in a premature stop codon (TGA) in exon 2 and disruption of the MARK2 gene in the near-haploid genome. (B) In the 5-KO MEF line, the MARK2 gene is disrupted with a CRISPR/Cas9-induced 1-bp insertion in exon 2, resulting in a premature stop codon (TGA) in exon 2 in both alleles of the gene. (C) In the 4-KO and 5-KO MEF lines, the PERK gene is disrupted with a CRISPR/Cas9-induced 1/2-bp deletion in exon 1, resulting in a premature stop codon (TGA or TAA) in exon 2 in both alleles of the gene. (D) In the 4-KO and 5-KO MEF lines, the HRI gene is disrupted with a CRISPR/Cas9-induced 1-bp or 4-bp deletion in exon 1, resulting in a premature stop codon (TAA) in exon 2 in both alleles of the gene. (E) In the 4-KO and 5-KO MEF lines, the GCN2 gene is disrupted with a CRISPR/Cas9-induced 13-bp or 6-bp deletion in exon 2, resulting in a premature stop codon (TGA or TAA) in exon 2 in both alleles of the gene. (F) In the 4-KO′ and 5-KO′ MEF lines, the existing PKR knockout allele (4-KO and 5-KO) is further edited using CRISPR to disrupt a remnant C-terminal fragment of PKR. In the original PKR knockout allele, exons 2 and 3 were replaced with a segment containing the N [file pbio.3001096.s002.tif]

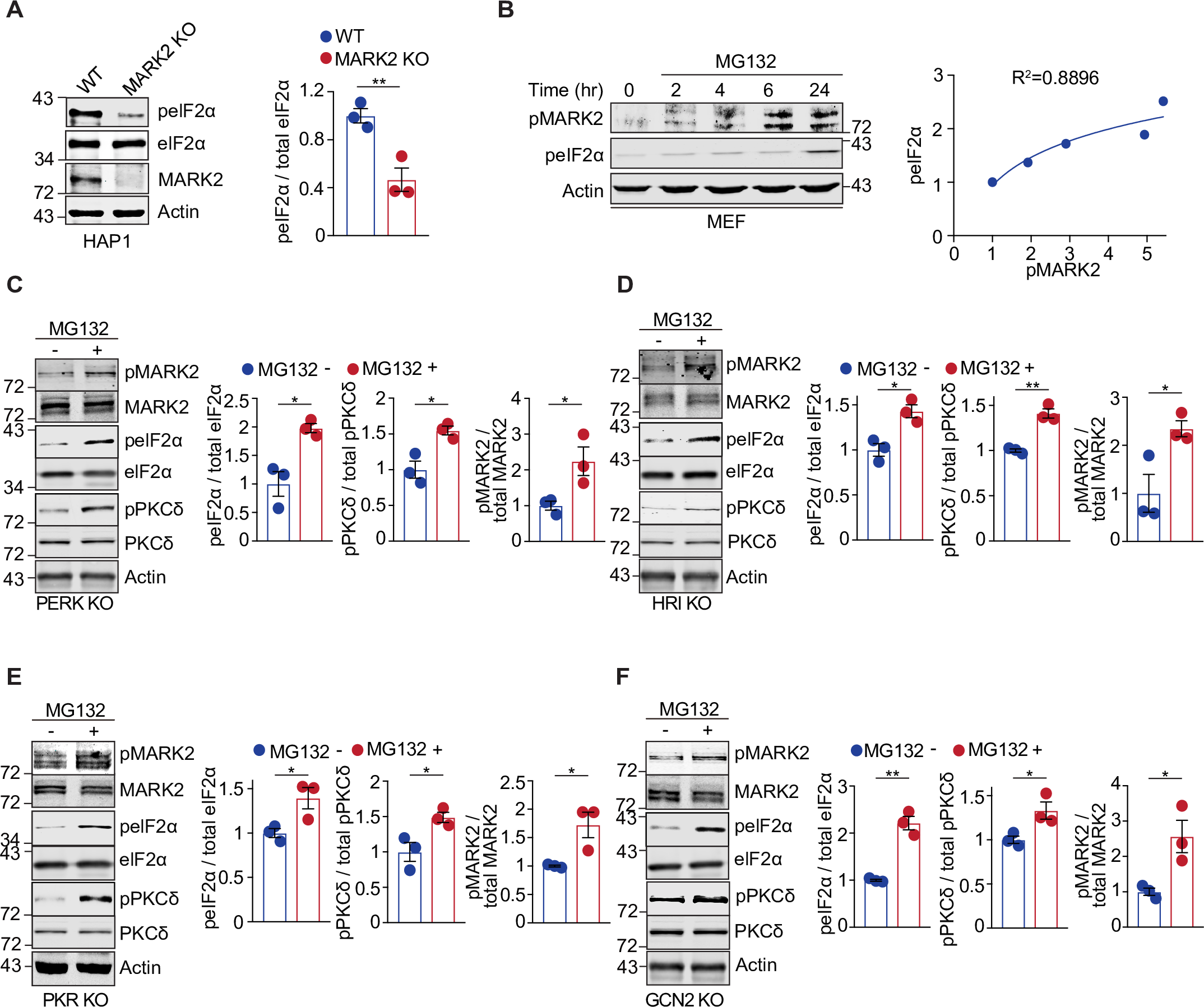

Supplement: S3 Fig — (A) Deletion of the MARK2 gene decreases the phosphorylation of eIF2α-51S in human HAP1 cells. Bar graph represents quantification of the immunoblots (n = 3). (B) Immunoblotting analyses of MEFs treated with the proteasome inhibitor MG132 (500 nM) indicate increased levels of phosphorylated eIF2α-51S that correlated with the levels of phosphorylated MARK2-595T over the 24-h time course of the MG132 treatment. The graph used to calculate the Pearson coefficient is shown to indicate the significant correlation (p = 0.0161). (C-F) Immunoblotting analyses of WT and knockout MEFs treated with MG132 indicate that the PKCδ-MARK2-eIF2α signaling pathway can be activated, as measured by the levels of phosphorylated eIF2α-51S, MARK2-595T, and PKCδ-505T independently of any of the previously known eIF2α kinases, including PERK, HRI, PKR, and GCN2. Bar graphs represent the quantification of the immunoblots (n = 3). Error bars represent ± SEM. *p ≤ 0.05; **p ≤ 0.01. The data underlying the figure can be found in S1 Data. eIF2α, eukaryotic initiation factor 2 alpha; GCN2, general control nonderepressible factor 2 kinase; HRI, heme-regulated eIF2α kinase; KO, knockout; MARK2, microtubule affinity-regulating kinase 2; MEF, mouse embryonic fibroblast; PERK, PKR-like ER-resident kinase; PKCδ, protein kinase C delta; PKR, protein kinase R; WT, wild-type. (TIF) [file pbio.3001096.s003.tif]

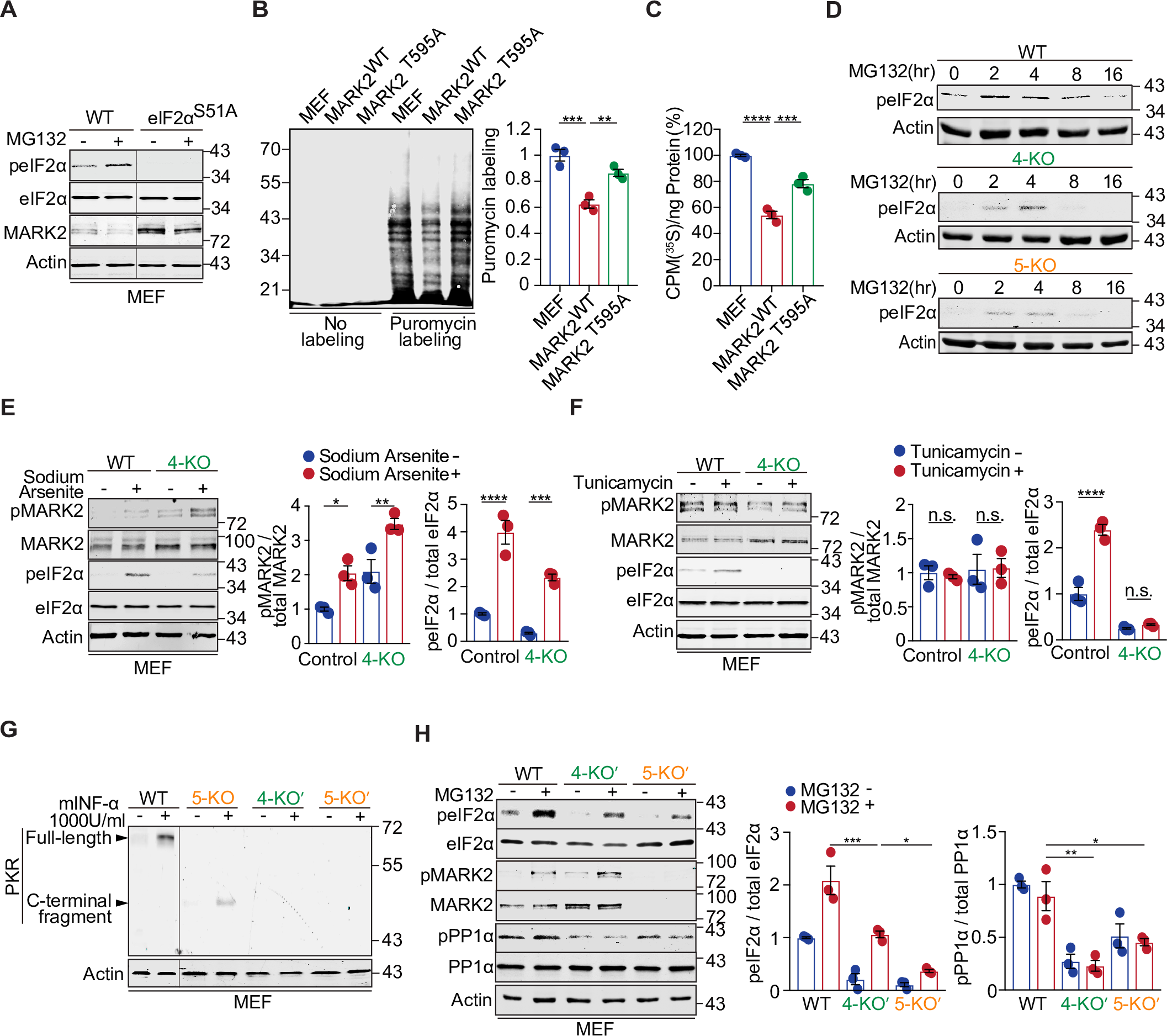

Supplement: S4 Fig — (A) The specificity of the antibody against phosphorylated eIF2α-51S was verified in an eIF2αS51A knockin mutant MEF line, in which the S51A mutation abolished the immunoblot signal of phosphorylated eIF2α-51S observed in WT MEFs treated with MG132. (B) MEFs stably overexpressing MARK2WT or MARK2T595A were pulsed-labeled with puromycin for 10 min, and the cell lysates were analyzed by SDS-PAGE and immunoblotting against puromycin-labeled proteins. The bar graph represents the quantification of the immunoblots (n = 3). (C) MEFs stably overexpressing MARK2WT or MARK2T595A were pulse-labeled with 35S-methionine and 35S-cysteine for 1 h, and the cell lysates were analyzed by liquid scintillation counting for 35S-labeled proteins (n = 3). The quantitative results indicate that overexpression of MARK2WT caused attenuation of global translation, while the T595A mutation impaired its regulatory activity. (D) A time course of the treatment with MG132 (20 μM) at indicated times shows that the phosphorylation of eIF2α-51S peaked around 4 h in the MEFs. (E) Immunoblot analyses of MEFs treated with sodium arsenite (200 μM, 1 h) indicate that the phosphorylation of MARK2-595T and eIF2α-51S was increased by the stress in WT and 4-KO cells. Bar graphs represent the quantification of the immunoblots (n = 3). (F) Immunoblot analyses of MEFs treated with tunicamycin (24 μg/ml, 2 h) indicate that the phosphorylation of MARK2-595T was not affected by the stress, while the phosphorylation of eIF2α-51S could be independently induced in WT but not the 4-KO cells. Bar graphs represent the quantification of the immunoblots (n = 3). (G) Immunoblot analyses of WT, 5-KO, 4-KO′, and 5-KO′ MEFs treated with mINF-α (1,000 U/ml for 18 h) indicate a remnant C-terminal fragment of PKR in 5-KO cells, which has been deleted in 4-KO′ and 5-KO′ cells as designed. (H) Immunoblot analyses of WT, 4-KO′, and 5- KO′ MEFs treated with MG132 indicate that eIF2α-51S is phosphorylated in response to the stress in [file pbio.3001096.s004.tif]

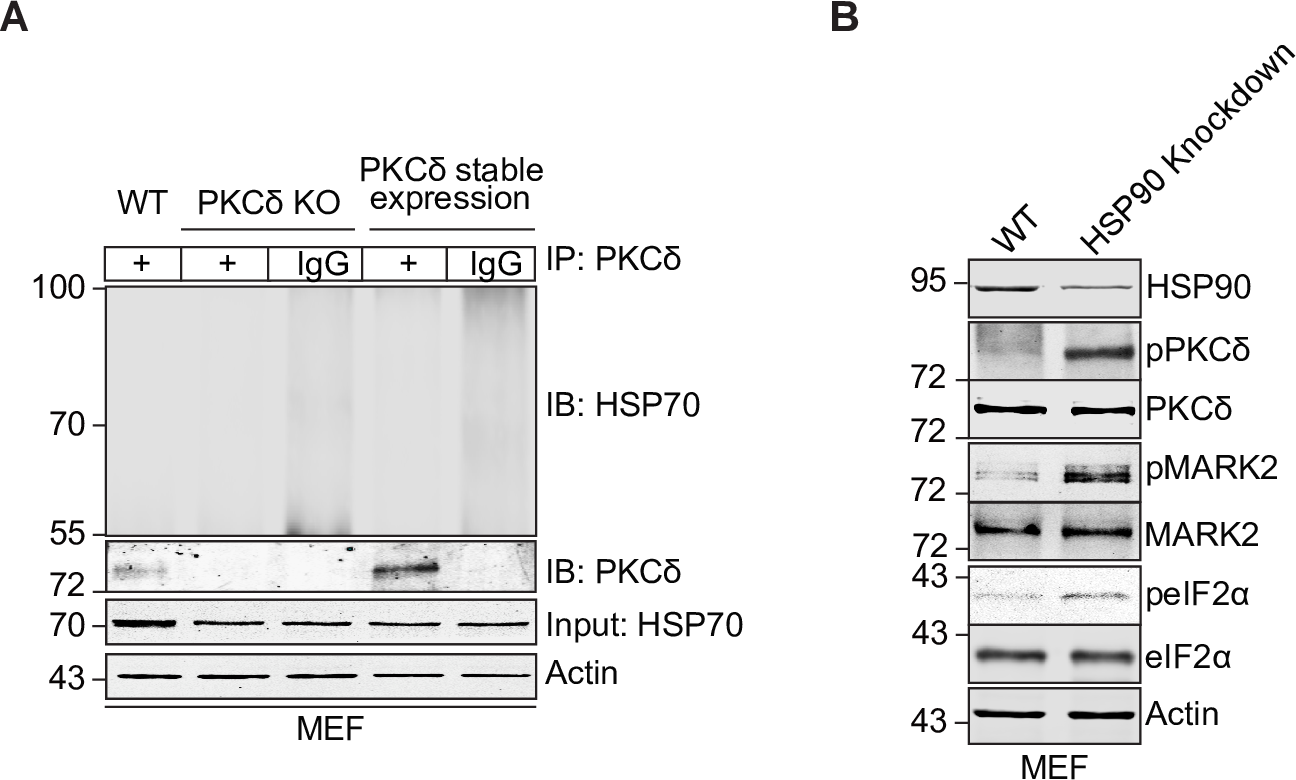

Supplement: S5 Fig — (A) In coimmunoprecipitation analyses, no HSP70 was detected in immunoprecipitates pulled down by the anti-PKCδ antibody from WT MEFs, those from PKCδ KO MEFs, or those from MEFs stably expressing PKCδ. IgG was used as a control for the anti-PKCδ antibody. (B) Comparison of WT MEFs and those with HSP90 knockdown by CRISPR in immunoblot analyses indicate that the down-regulation of HSP90 substantially increased the phosphorylation of PKCδ-505T, MARK2-595T, and eIF2α-51S. eIF2α, eukaryotic initiation factor 2 alpha; HSP70, heat shock protein 70; HSP90, heat shock protein 90; IgG, immunoglobulin G; IB, immunoblotting; IP, immunoprecipitation; KO, knockout; MARK2, microtubule affinity-regulating kinase 2; MEF, mouse embryonic fibroblast; PKCδ, protein kinase C delta; WT, wild-type. (TIF) [file pbio.3001096.s005.tif]

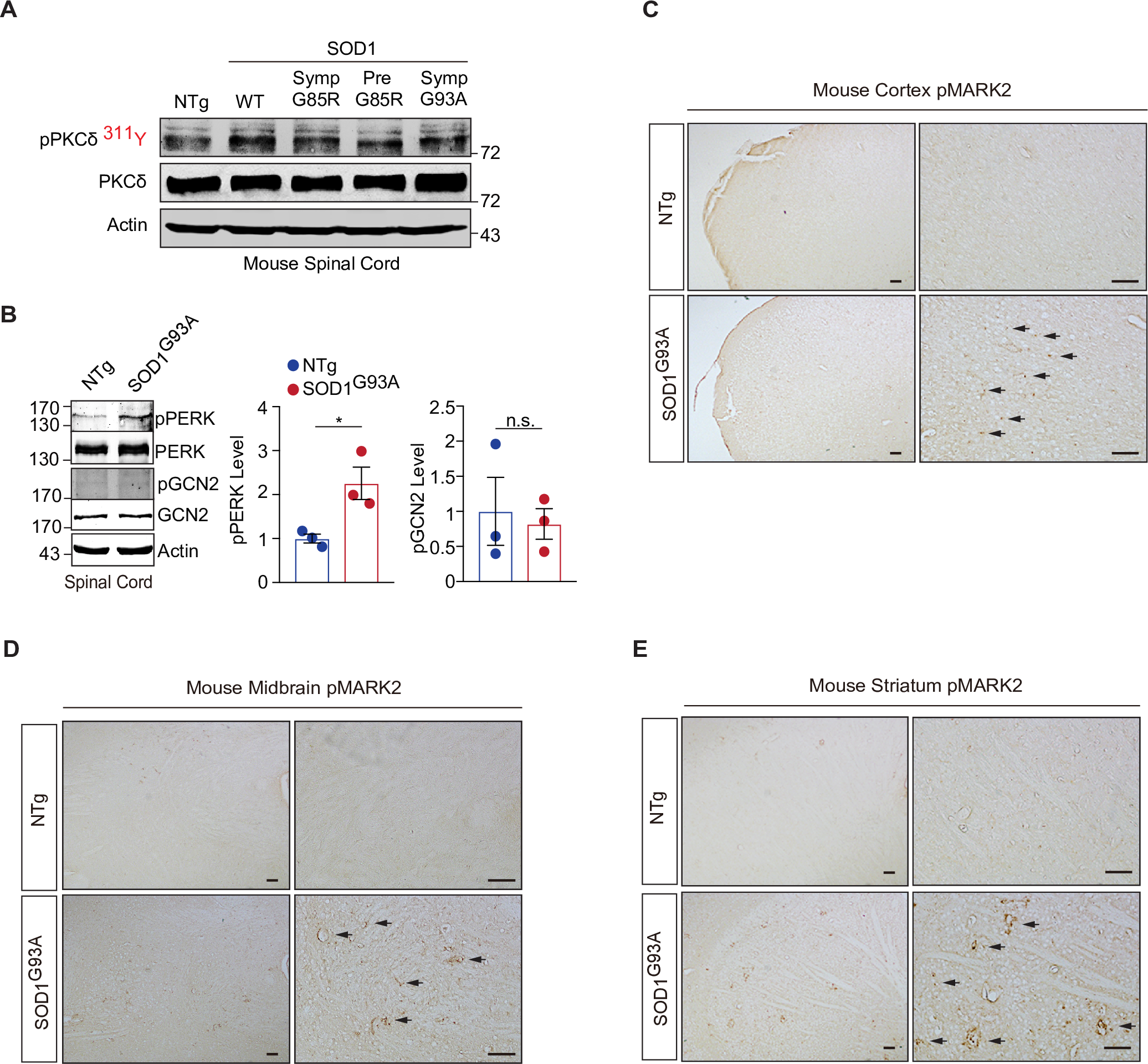

Supplement: S6 Fig — (A) Immunoblot analyses of spinal cord lysates from NTg, SOD1WT-YFP, presymptomatic and symptomatic SOD1G85R-YFP, and SOD1G93A transgenic mice show no change in the levels of phosphorylation of PKCδ at tyrosine 311. (B) Immunoblot analyses of spinal cords from symptomatic SOD1G93A mice and NTg littermate controls indicate that the level of phosphorylated PERK-980T was significantly increased in the SOD1G93A mice, while no change was detected for GCN2. Immunohistochemical analyses of phosphorylated MARK2 in the brain cortex (C), midbrain (D), and striatum (E) from symptomatic SOD1G93A transgenic mice and NTg controls. The staining of phosphorylated MARK2-595T in all 3 brain regions is increased in the SOD1G93A mice as compared to NTg mice. Error bars represent ± SEM. *p ≤ 0.05. Scale bar: 50 μm. The data underlying the figure can be found in S1 Data. ALS, amyotrophic lateral sclerosis; GCN2, general control nonderepressible factor 2 kinase; MARK2, microtubule affinity-regulating kinase 2; n.s., nonsignificant; NTg, nontransgenic; PERK, PKR-like ER-resident kinase; PKCδ, protein kinase C delta; Pre, presympomatic; SOD1, Cu/Zn superoxide dismutase; Symp, symptomatic; WT, wild-type. (TIF) [file pbio.3001096.s006.tif]

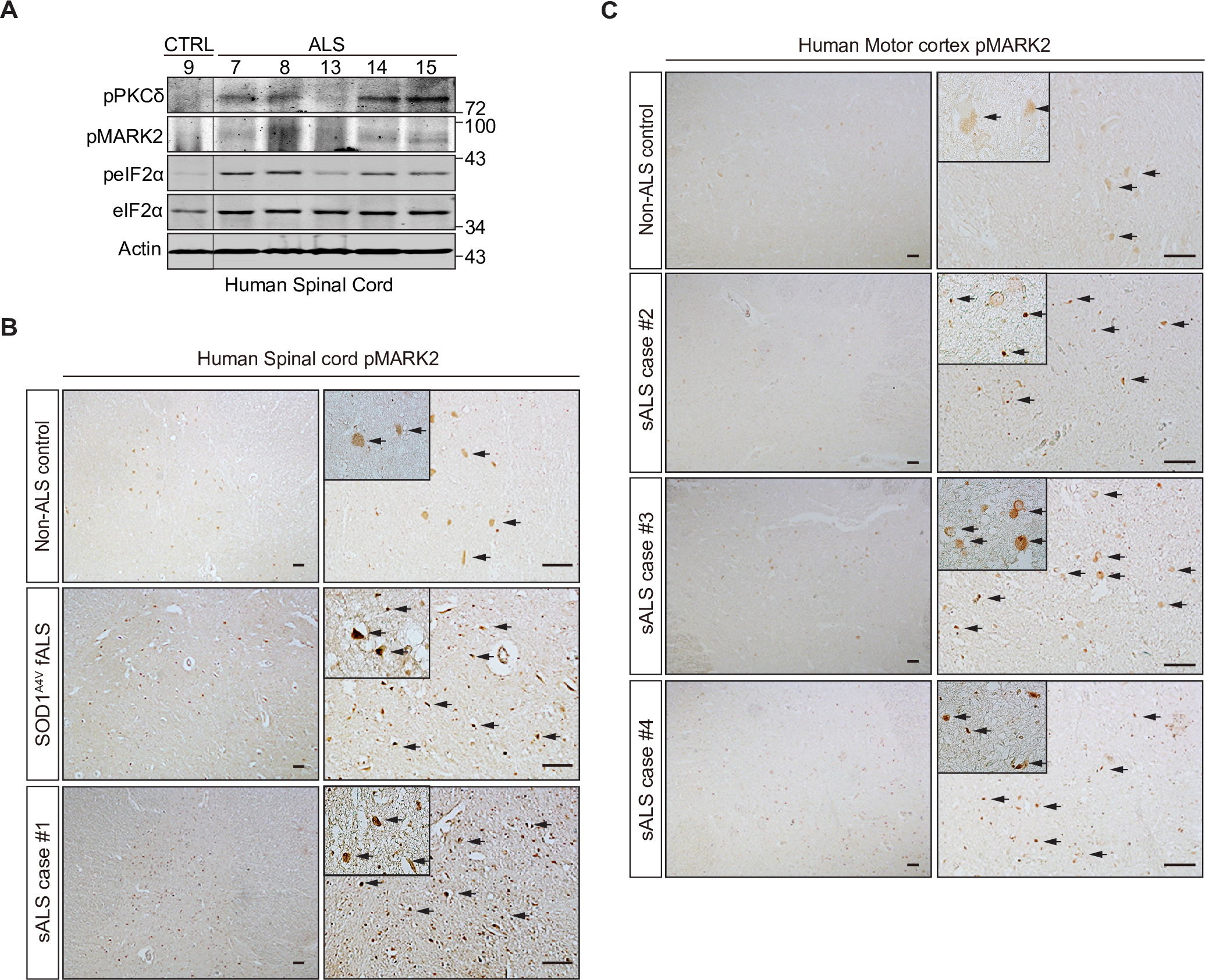

Supplement: S7 Fig — (A) Representative immunoblot analyses of PKCδ, MARK2-595T, and eIF2α in the spinal cord tissues from ALS patients and non-ALS controls, indicating that increased phosphorylation of PKCδ-505T, MARK2-595T, and eIF2α-51S is a general phenotype in patient tissues. (B) Immunohistochemical staining of phosphorylated MARK2-595T in the spinal cords from an SOD1A4V-ALS patient, an sALS patient, and a non-ALS age-matched control case. (C) Immunostaining for phosphorylated MARK2-595T in the motor cortex of 3 different sporadic ALS patients. Increased phosphorylation of MARK2-595T is observed in all ALS patient tissues. Scale bar: 50 μm. ALS, amyotrophic lateral sclerosis; CTRL, control; eIF2α, eukaryotic initiation factor 2 alpha; fALS, familial ALS; MARK2, microtubule affinity-regulating kinase 2; PKCδ, protein kinase C delta; sALS, sporadic ALS; SOD1, Cu/Zn superoxide dismutase. (TIF) [file pbio.3001096.s007.tif]

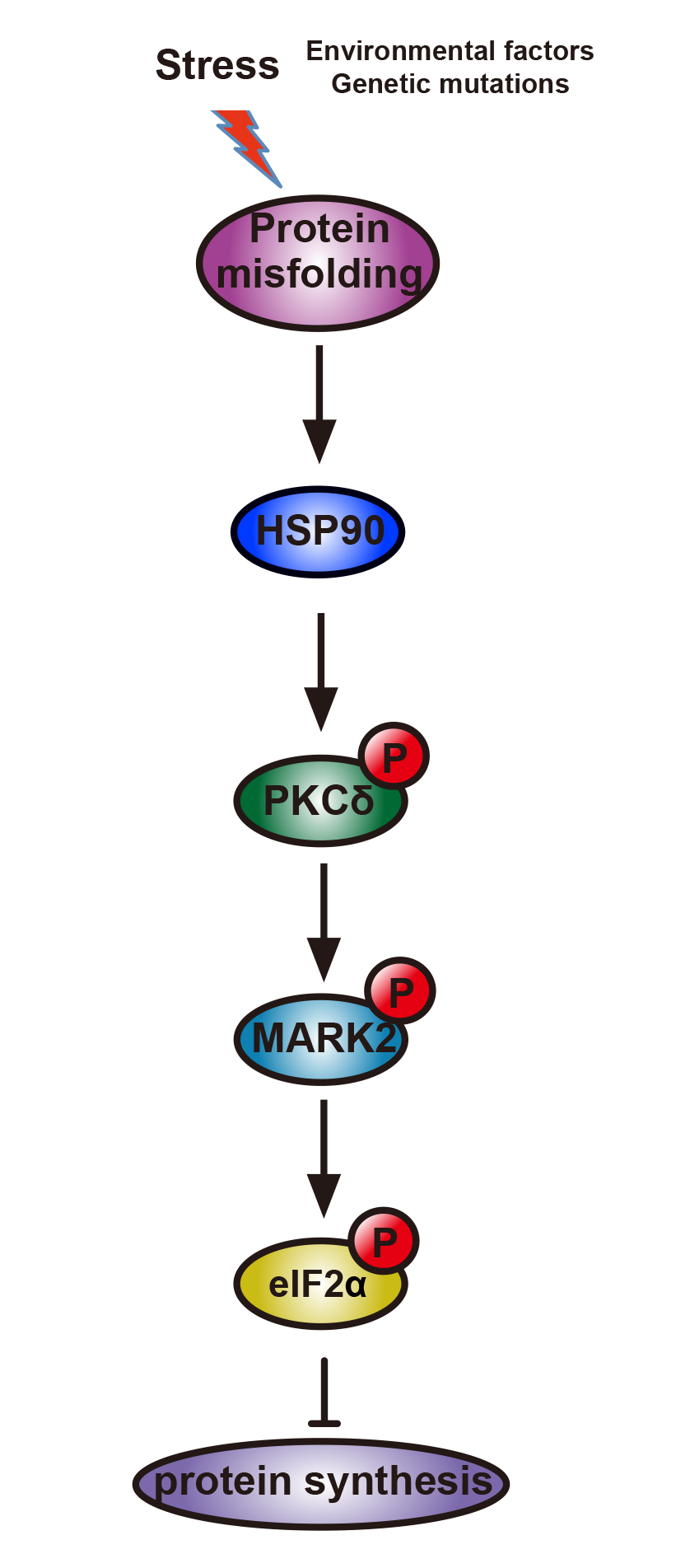

Supplement: S8 Fig — Upon protein misfolding stress, HSP90 is sequestered by misfolded proteins, resulting in phosphorylation and activation of PKCδ, which in turn activates MARK2 that phosphorylates eIF2α. The increased phosphorylation of eIF2α leads to translational attenuation. eIF2α, eukaryotic initiation factor 2 alpha; HSP90, heat shock protein 90; MARK2, microtubule affinity-regulating kinase 2; PKCδ, protein kinase C delta. (TIF) [file pbio.3001096.s008.tif]

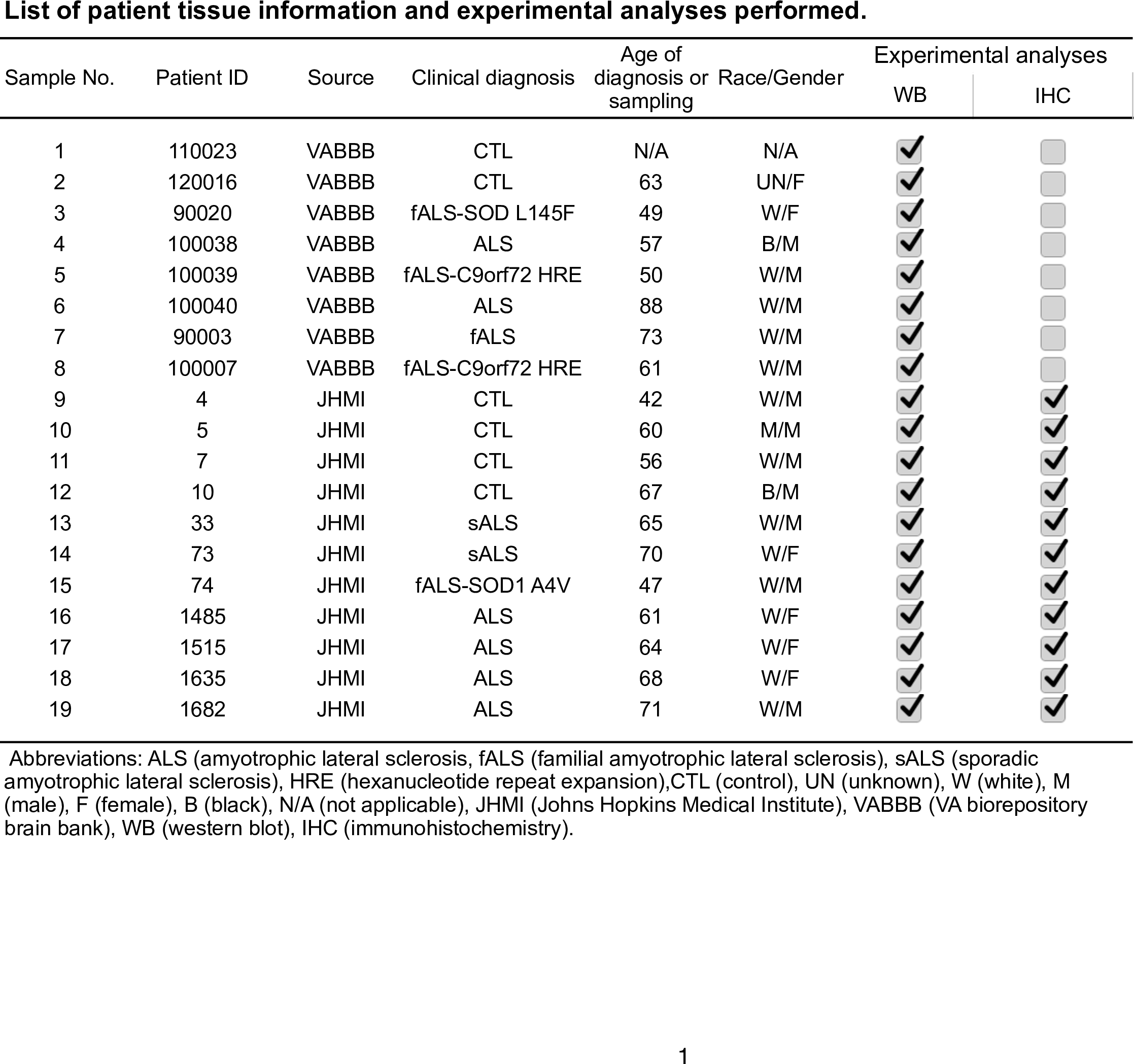

Supplement: S1 Table — (TIF) [file pbio.3001096.s009.tif]
