# Supplementary material for: MARK2 phosphorylates eIF2α in response to proteotoxic stress
Source: PLoS Biol. 2021 Mar 11;19(3):e3001096. doi: 10.1371/journal.pbio.3001096 (PMC7951919; doi:10.1371/journal.pbio.3001096)

Fig. 1A

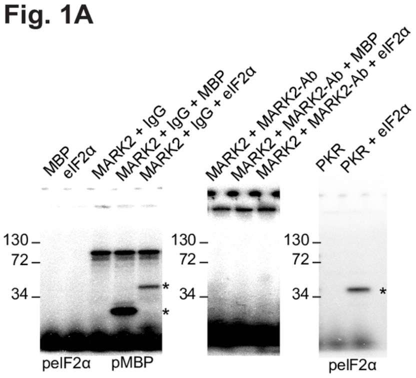

Fig. 1B

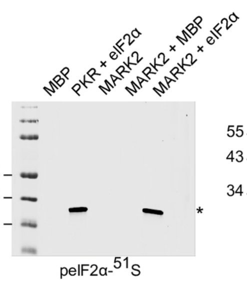

Fig. 1D

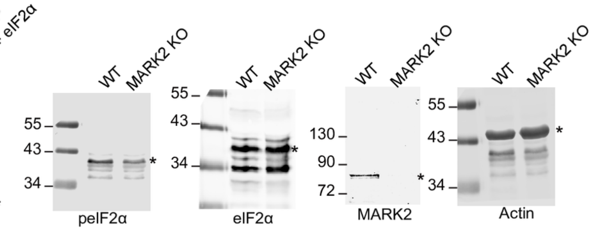

Fig. 1E

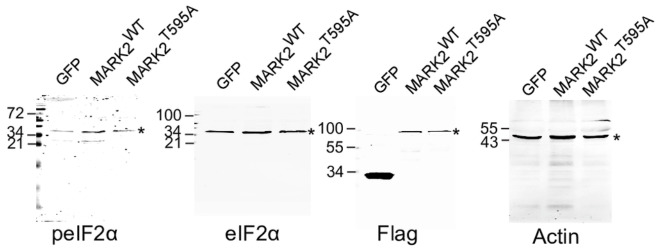

Fig. 1F

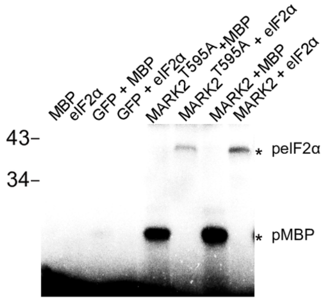

Fig. 2A

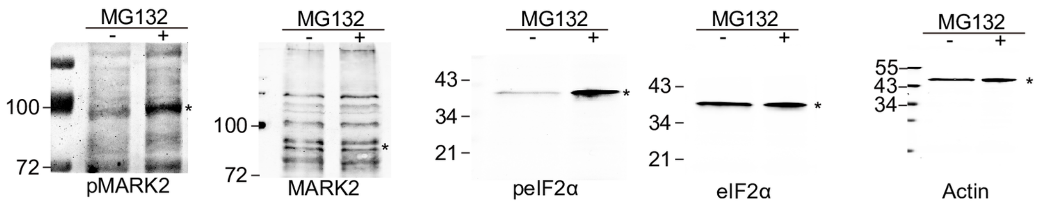

Fig. 2B

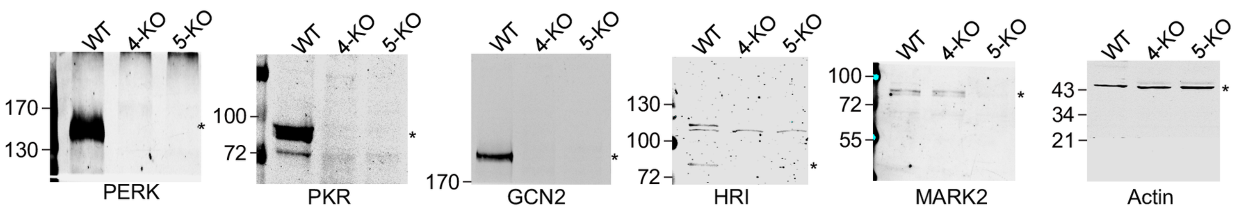

Fig. 2C

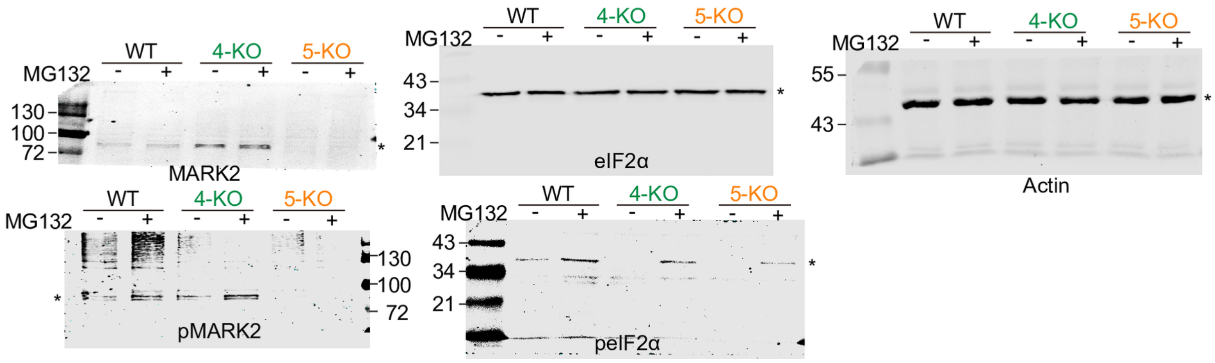

Fig. 2D

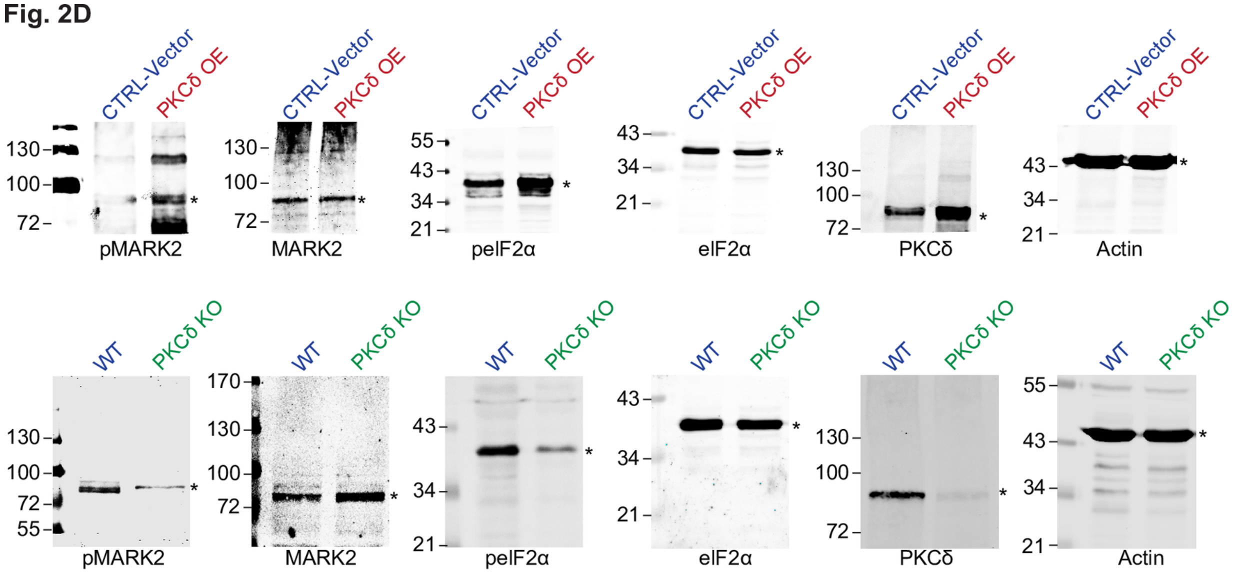

Fig. 2E

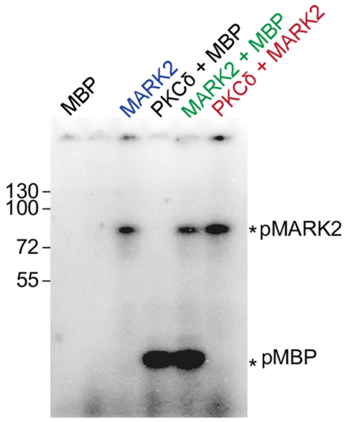

Fig. 2F

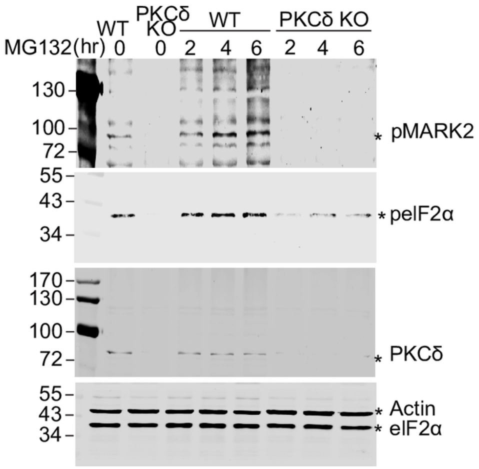

Fig. 2G

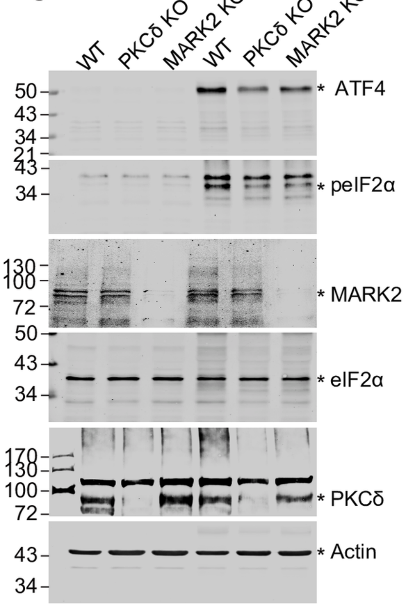

Fig. 3A

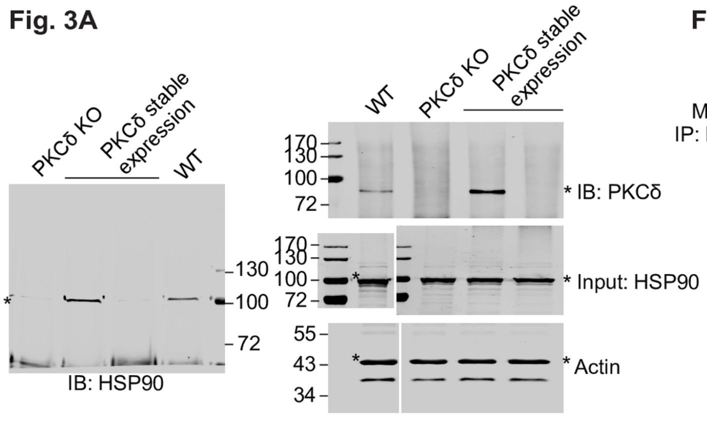

Fig. 3B

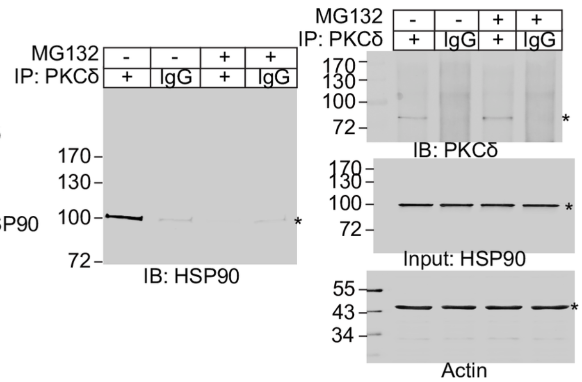

Fig. 3C

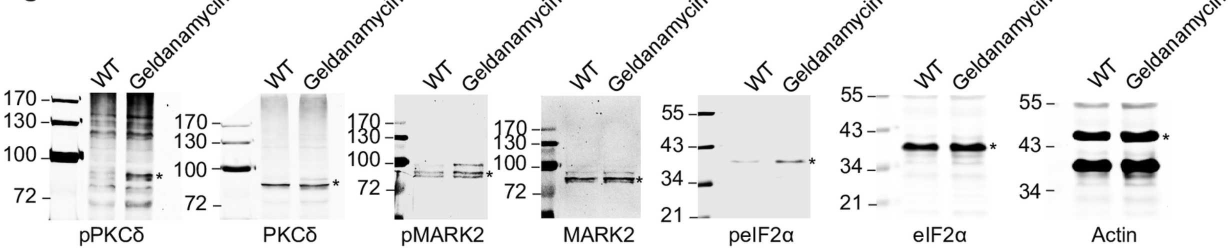

Fig. 4A

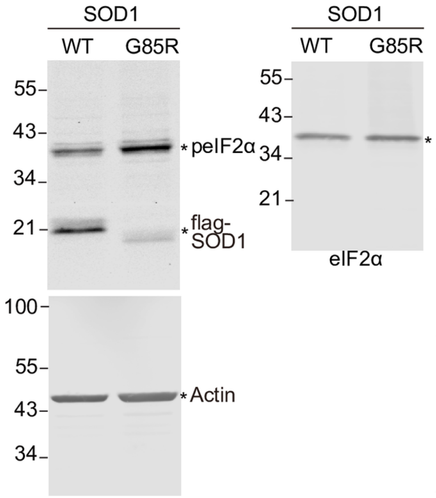

Fig. 4B

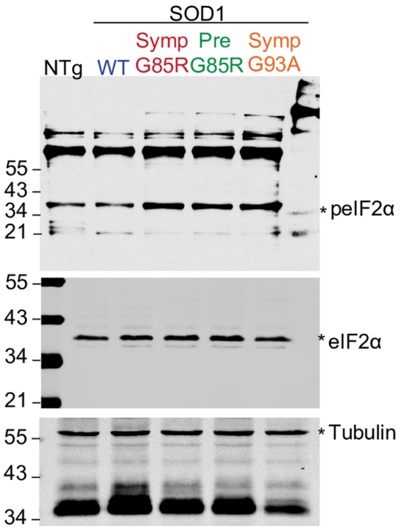

Fig. 5A

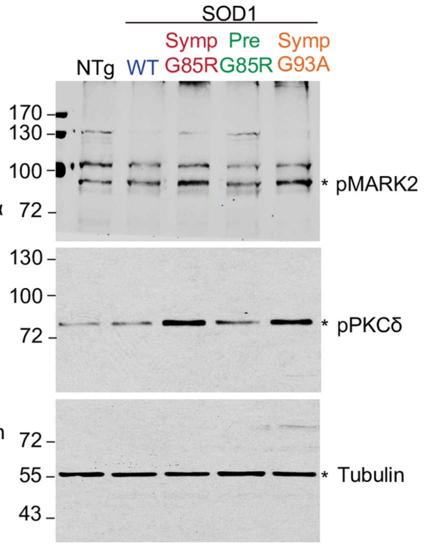

Fig. 5D

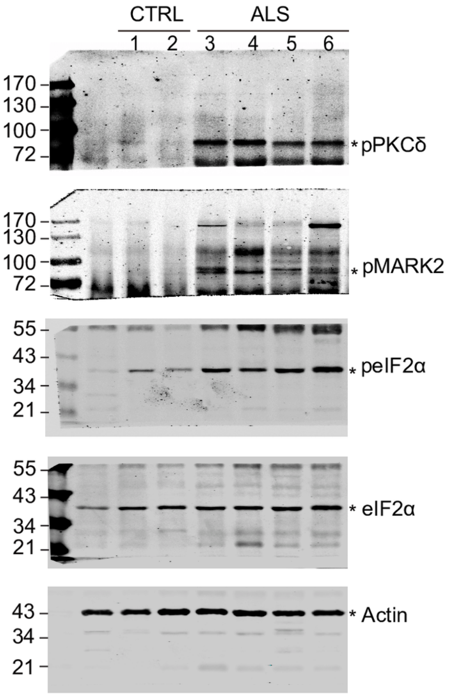

Supplemental Fig. 1A

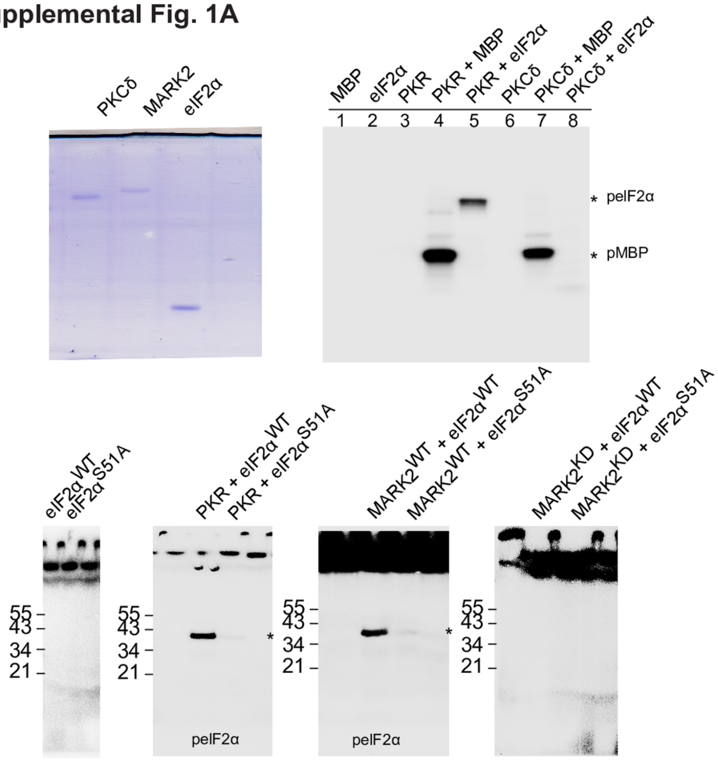

Supplemental Fig. 3A

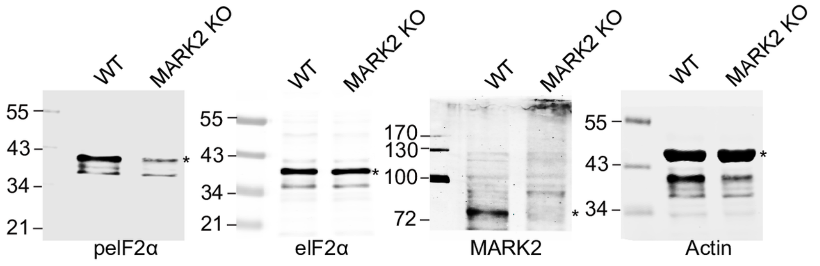

Supplemental Fig. 3B

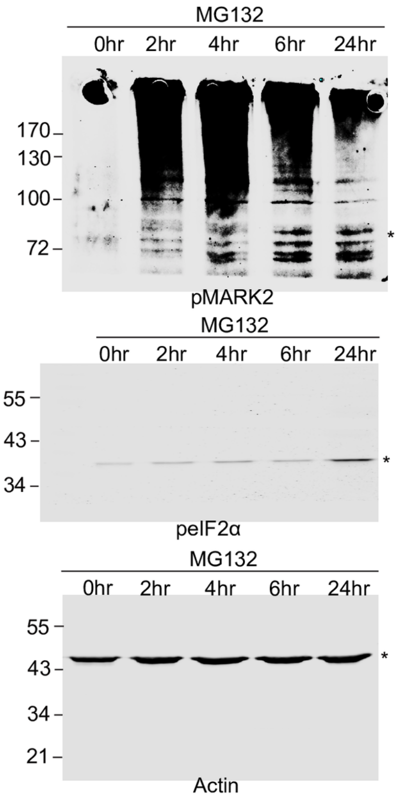

Supplemental Fig. 3C

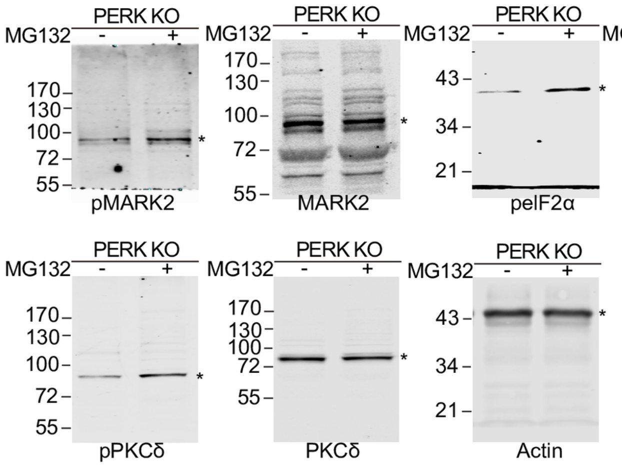

Supplemental Fig. 3D

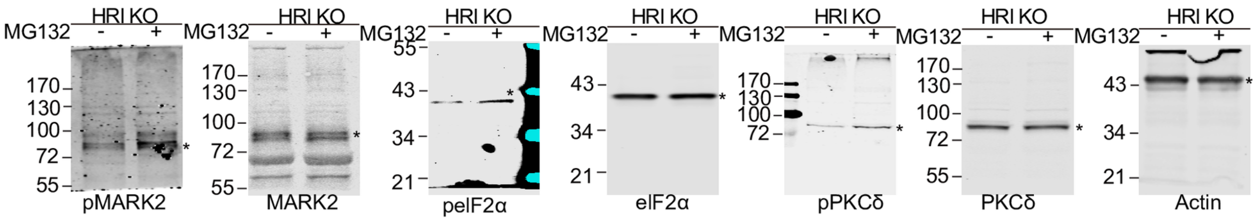

Supplemental Fig. 3E

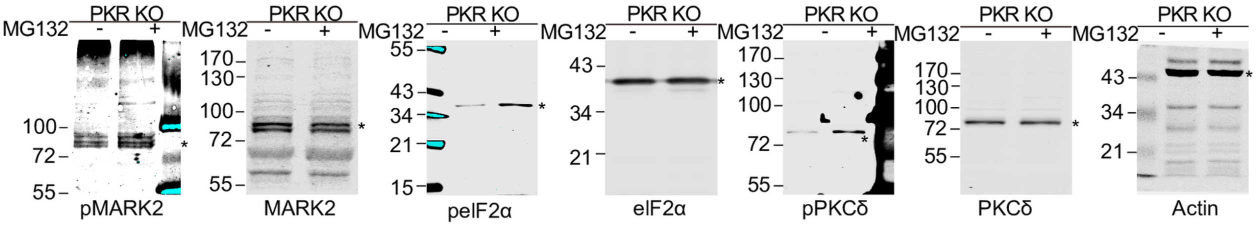

Supplemental Fig. 3F

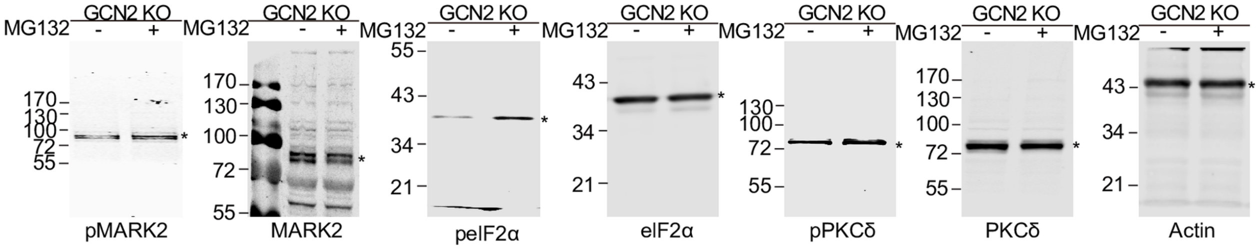

Supplemental Fig. 4A

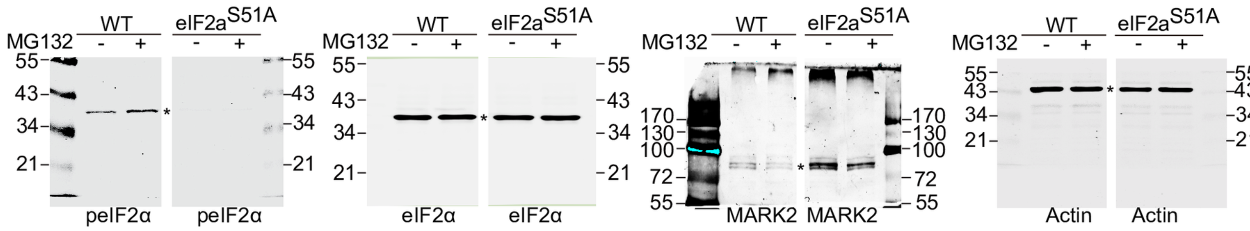

Supplemental Fig. 4B

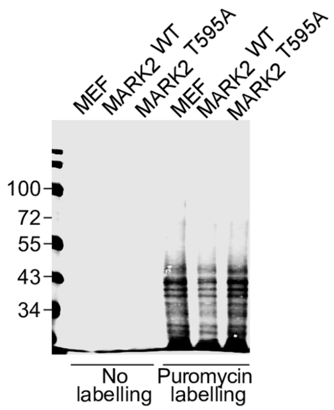

Supplemental Fig. 4D

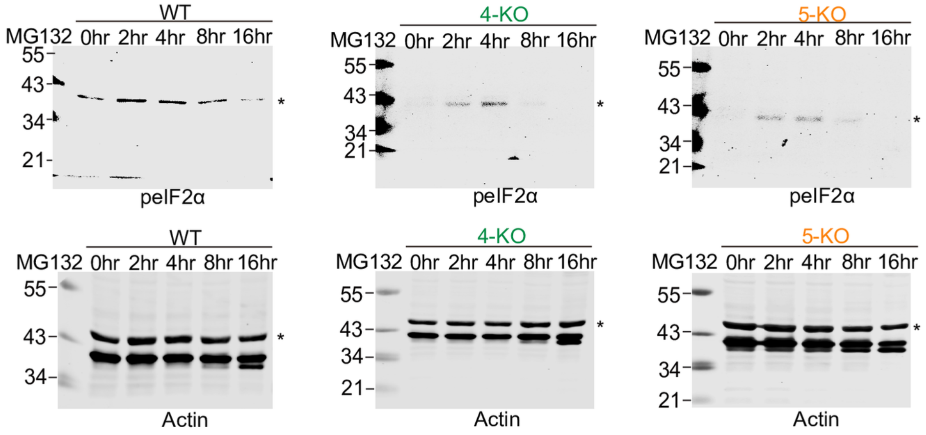

Supplemental Fig. 4E

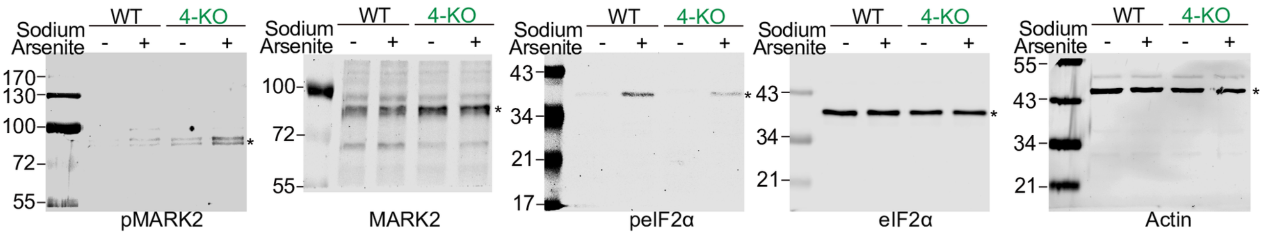

Supplemental Fig. 4F

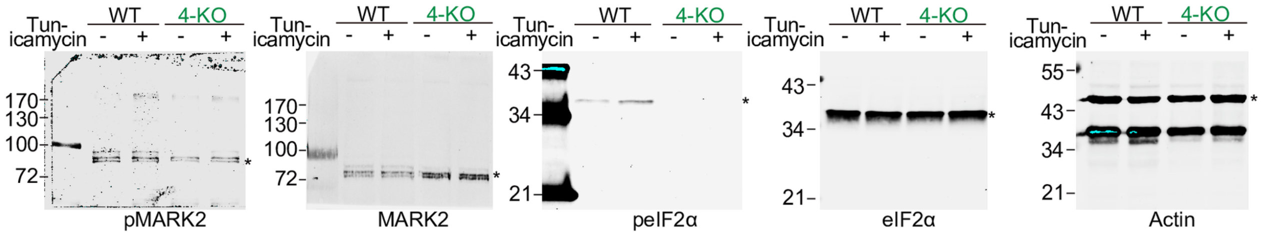

Supplemental Fig. 4G

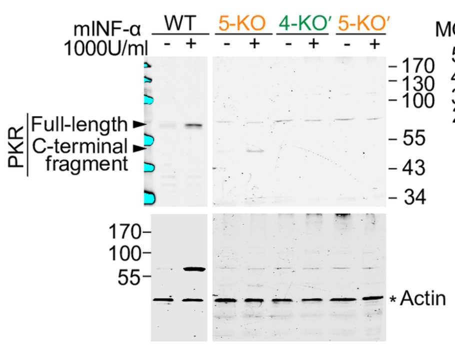

Supplemental Fig. 4H

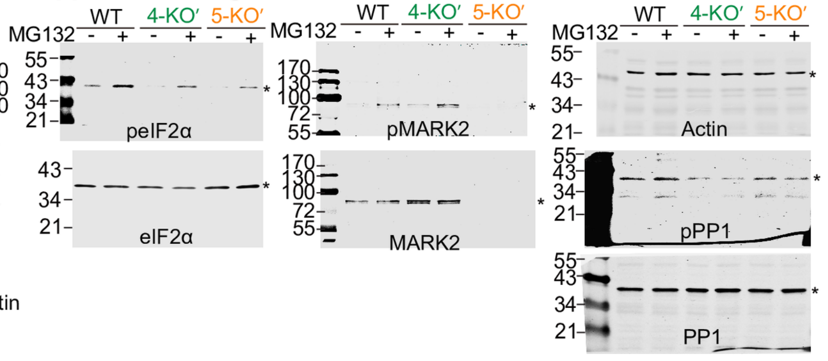

Supplemental Fig. 5A

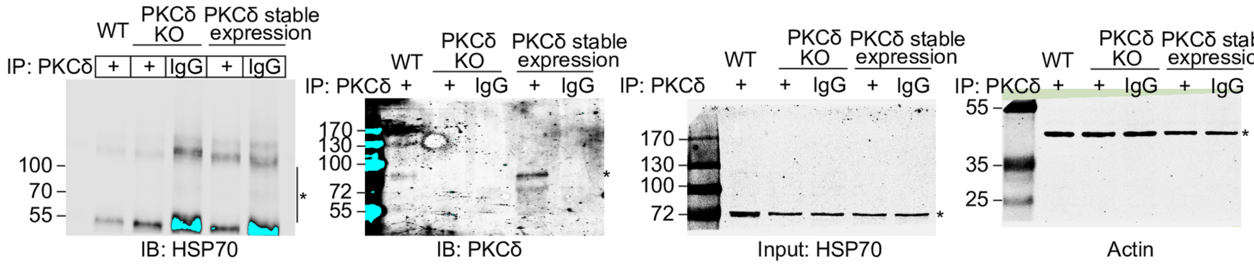

Supplemental Fig. 5B

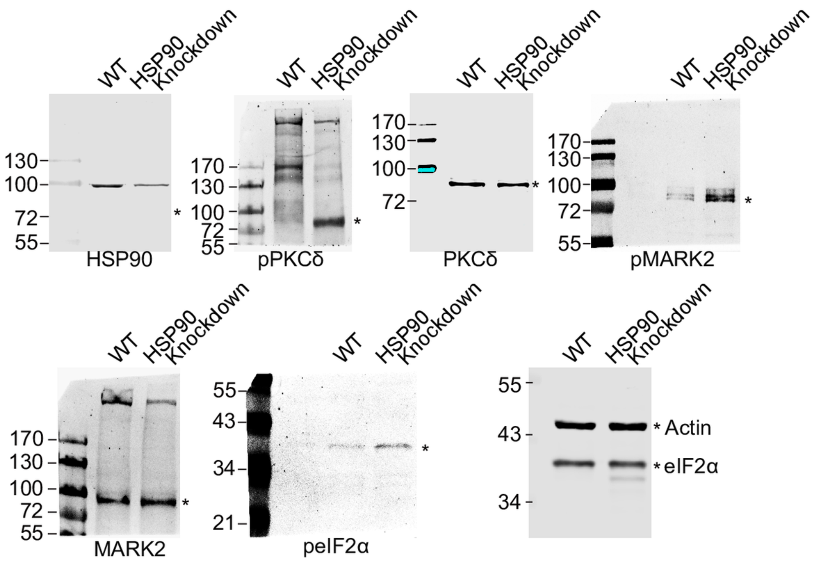

Supplemental Fig. 6A

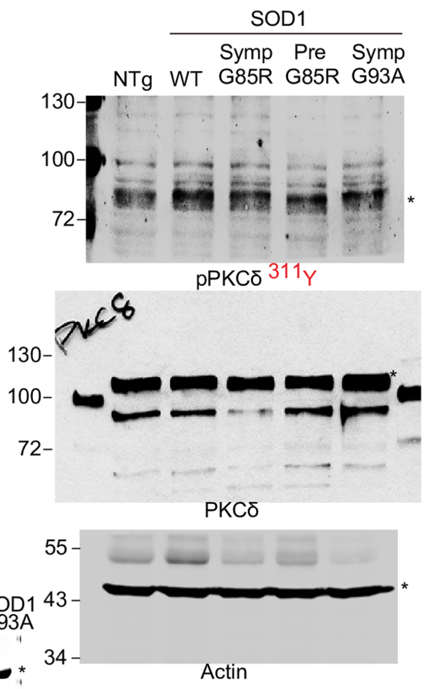

Supplemental Fig. 6B

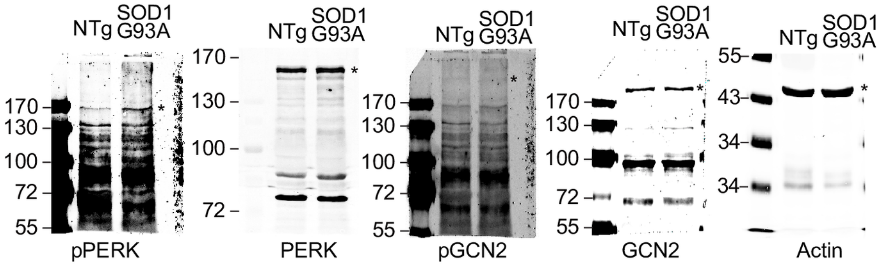

Supplemental Fig. 7A

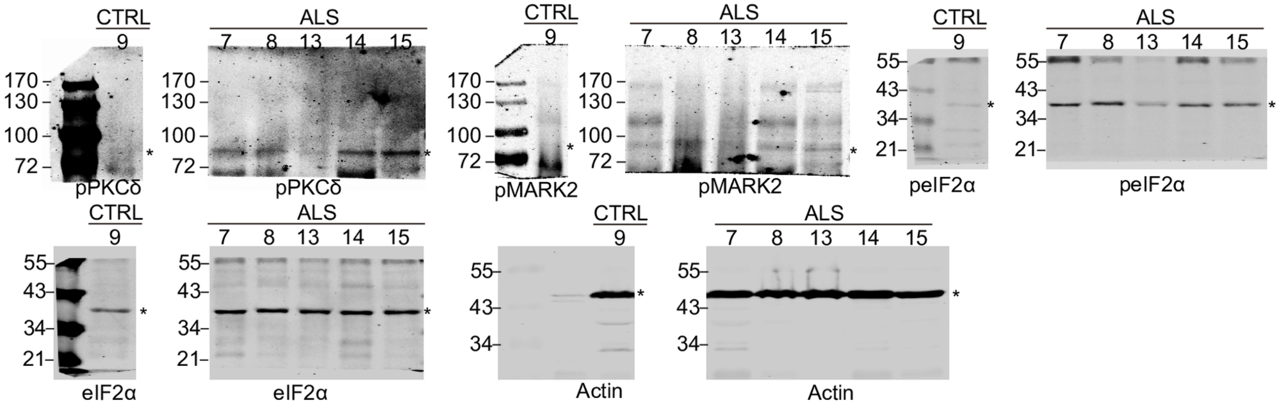

Supplement: S1 Raw Images — (PDF) [file pbio.3001096.s011.pdf]
